# A Blast‐Resistant NLR Gene Confers Drought Resistance by Competitively Interacting with an E3 Ligase to Protect Phenylalanine Ammonia‐Lyase in Rice

**DOI:** 10.1002/advs.202502662

**Published:** 2025-07-21

**Authors:** Denghao Xiang, Haifu Tu, Yang Yuan, Yilong Yao, Wanwen Liao, Huaijun Wang, Yu Yan, Yao Wang, Yu Chen, Di Liu, Qingya Lv, Haidong He, Honghong Hu, Xuelei Lai, Meng Yuan, Haiyan Xiong, Faming Dong, Lizhong Xiong

**Affiliations:** ^1^ National Key Laboratory of Crop Genetic Improvement and National Center of Plant Gene Research (Wuhan) Huazhong Agricultural University Wuhan 430070 China; ^2^ Hubei Hongshan Laboratory Wuhan 430070 China; ^3^ Yazhouwan National Laboratory Sanya 572024 China

**Keywords:** drought, GWAS, genetic improvement, NLR, rice

## Abstract

Drought and diseases represent major challenges to achieve high yield in crops, underscoring the urgent need to explore drought‐ and disease‐resistant genetic resources and breed crop varieties with multi‐stress resistance. Here, using a genome‐wide association study combined with function analyses, *PibH8*, a homologous of the blast resistance gene *Pib* encoding a nucleotide‐binding leucine‐rich repeat receptor (NLR) is identified, which plays a crucial role in rice drought resistance. PibH8 interacts with phenylalanine ammonia‐lyase OsPAL1, a rate‐limiting enzyme in phenylpropanoids biosynthesis pathway in rice. It protects OsPAL1 from degradation by competitively binding to E3 ubiquitin ligase OsFBK16, which facilitates OsPAL1 degradation. This protective mechanism enhances PAL activity, leading to increased lignin and flavonoids content and improved drought resistance. Genetic evidence indicates that *PibH8* acts upstream of *OsPAL1* in conferring drought resistance. Furthermore, a causal variation in the *PibH8* promoter that is associated with drought resistance is identified. Introgression of a superior haplotype, which exhibits high *PibH8* expression, into the elite rice variety Kongyu131 significantly improved drought and blast resistance. This research not only elucidates a regulatory mechanism of NLR protein in drought resistance, but also highlights a promising breeding value of *PibH8* for simultaneously improving drought and blast resistance.

## Graphical abstract

The NLR protein PibH8 interacts with phenylalanine ammonia‐lyase OsPAL1 and E3 ubiquitin ligase OsFBK16. Through competitive binding, PibH8 weakens the interaction between OsFBK16 and OsPAL1, thereby reducing the degradation of OsPAL1 by OsFBK16. The Hap1 allele of *PibH8* exhibits a higher expression level than Hap2, thus accumulating more PibH8 protein, which in turn promotes greater accumulation of OsPAL1 and higher levels of lignin and flavonoids, thereby enhancing drought and disease resistance.

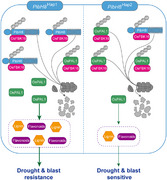

## 1 Introduction

Drought is a major challenge to agricultural production, causing annual crop yield losses greater than all pathogens combined.^[^ ^1^ ^]^ Rice, providing the staple food for half of the global population, faces severe growth and yield restrictions due to drought under frequent extreme climate.^[^ ^2^ ^]^ Consequently, it is imperative to elucidate the genetic and molecular mechanisms of drought resistance in rice and to breed drought‐resistant varieties. Drought resistance is a complex trait characterized by various physiological and molecular responses influenced by numerous alleles with minor effect.^[^ ^3^ ^]^ Recent applications of genome‐wide association study (GWAS) have successfully identified several drought‐related genes in rice, such as *OsPP15*, *DROT1*, and *DRG9*.^[^ ^4^ ^]^ However, the successful integration of these findings into effective drought‐resistant breeding programs remains limited. Additionally, diseases like blast continue to threaten rice production, and abiotic and biotic stresses frequently occurs sequentially or simultaneously in field. Despite many disease resistance genes have been reported in rice, very few have been demonstrated with breeding value for improving both drought and blast resistance.

Nucleotide‐binding site (NBS) leucine‐rich repeat (LRR) receptors (NLRs), representing the largest class of plant disease resistance proteins, activate a robust defense response known as effector‐triggered immunity (ETI).^[^ ^5^ ^]^ NLRs typically comprise three domains: an NB‐ARC (nucleotide‐binding adaptor shared by Apaf1, certain R genes and CED4) domain, a LRR domain, and an N‐terminal TIR (toll‐interleukin‐1 receptor) or CC (coiled‐coil) domain. The N‐terminal CC domain is critical for protein‐protein interactions,^[^ ^6^ ^]^ while the TIR domain is primarily involved in the recognition of effector or pathogen proteins.^[^ ^7^ ^]^ The NB‐ARC domain, also referred to as the NBS domain, is crucial for nucleotide binding,^[^ ^8^ ^]^ and the LRR domain is responsible for recognition specificity of NLR proteins, also regarded as a receptor structural domain.^[^ ^9^ ^]^ In rice, several NLRs have been reported to control disease resistance. *Pib* is the first NLR gene cloned by map‐based cloning strategy for blast resistance.^[^ ^10^ ^]^ Pib functions through the CC domain to facilitate homodimerization, while SH3 DOMAIN‐CONTAINING PROTEIN 2 (OsSH3P2) can inhibit Pib homodimerization in the absence of the effector *AvrPib*.^[^ ^11^ ^]^ Recently, a Pib homolog, PibH8, was found to interact with Pib and coordinately regulate blast resistance, and OsSH3P2 can also inhibit PibH8 homodimerization.^[^ ^12^ ^]^ Furthermore, a pair of NLR genes, *PigmR* and *PigmS*, have been demonstrated to antagonistically regulate rice blast resistance, in which, PigmS disrupts PigmR homodimerization, thus inhibiting rice blast resistance.^[^ ^13^ ^]^ Additionally, PigmR promotes the nuclear accumulation of RRM transcription factor PIBP1 and its homologous proteins to positively regulates blast resistance in rice.^[^ ^14^ ^]^ Blast fungal effectors, including AvrPi9, are capable of degrading PICI1 (PigmR‐interacting and chitin‐induced protein 1), which activates methionine‐mediated immunity by deubiquitinating K48‐ and K63‐linked ubiquitin and stabilizing methionine synthetases. The immune‐active PigmR interferes with the AvrPi9‐PICI1 interaction, preventing PICI1 degradation.^[^ ^15^ ^]^ Despite numerous studies highlighting the important role of NLRs in disease resistance, reports of NLRs involved in abiotic stress resistance remains scarce. The mutation of *Chilling sensitive 3*, which encodes an NLR protein, has been shown to enhance freezing tolerance in Arabidopsis.^[^ ^16^ ^]^ The gene *NLR4* was shown to be involved in the response to salt stress in Arabidopsis.^[^ ^17^ ^]^ In rice, *OsPi304* has been reported to respond to cold stress during the seedling stage.^[^ ^18^ ^]^ However, whether NLR genes are involved in drought response and how NLRs regulate abiotic stress resistance remain largely unknown.

Phenylalanine ammonia‐lyase (PAL) is the first rate‐limiting enzyme in phenylpropanoid biosynthesis pathway, facilitating the conversion of L‐Phe from the primary metabolic pool to the synthesis of trans‐cinnamic acid (t‐CA).^[^ ^19^ ^]^ Lignin and flavonoids, modulated by PALs, are two important products in the phenylpropanoid metabolism pathway and play significant roles in response to biotic and abiotic stresses.^[^ ^20^ ^]^ The activity of PAL is regulated at various levels. At the transcription level, various transcription factors have been reported to regulate *PALs* expression. In tobacco, NtMYBAS1, specifically expressed in the anther, binds to the MYB‐binding site in the *NtPAL1* promoter, positively regulating *NtPAL1* expression and phenylpropanoid synthesis.^[^ ^21^ ^]^ In rice, OsMYB30 directly binds to the promoter of *OsPAL6* and *OsPAL8*, positively regulating their expression and enhancing brown planthopper resistance.^[^ ^22^ ^]^ At the protein level, three E3 ubiquitin ligases (KFB01, KFB20, and KFB50) have been identified to mediate the ubiquitination‐mediated degradation of four PAL family members in Arabidopsis.^[^ ^23^ ^]^ In rice, OsFBK16 interacts with OsPAL1‐7, promoting their degradation and negatively regulating rice blast resistance.^[^ ^24^ ^]^ However, how PALs are regulated under abiotic stress conditions remains unclear.

In this study, we performed genetic analysis of drought resistance and focused on a quantitative trait locus, *Rice Drought Resistance 8* (*qRDR8*). A candidate causal gene, *PibH8*, was identified to confer drought resistance. We found that PibH8 interacts with OsPAL1, a phenylalanine ammonia‐lyase protein, which also confers drought resistance in rice. Further study showed that PibH8 protects OsPAL1 from degradation by disrupting the interaction between E3 ubiquitin ligase OsFBK16 and OsPAL1, leading to increased contents of lignin and flavonoids, thereby enhancing drought resistance. Furthermore, the introgression of the superior *PibH8* haplotype (Hap1) from the *indica* (*xian*) variety Huanghuazhan (HHZ) into the widely cultivated *japonica* (*geng*) variety Kongyu131 (KY131) (Hap.2) resulted in significant improvements in both drought resistance and blast resistance in the field. Our findings unveil a previously unreported NLR‐mediated mechanism for drought resistance and highlight the promising value of the superior *PibH8* haplotype for developing rice varieties with enhanced drought and blast resistance.

## 2 Results

### 2.1 GWAS of Drought Resistance and Identification of the Candidate Gene *PibH8*

**Figure 1:**
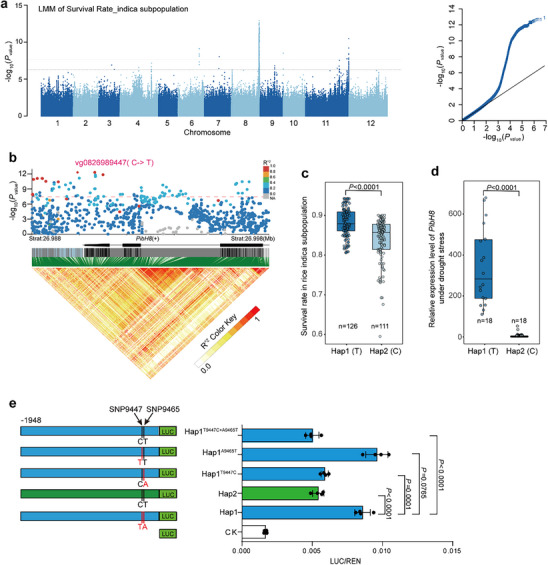
GWAS of seedling survival rate and the association analysis of the candidate gene *PibH8*. a) Manhattan plot of the GWAS under the standard mixed linear model (MLM) of seedling survival rate. The dashed horizontal line depicts the Bonferroni‐adjusted suggestive threshold (*p* = 6.54 × 10^−7^) and significant threshold (*p* = 3.27 × 10^−8^). b) *PibH8*‐based association mapping and pairwise LD analysis. The most significant SNP in the promoter of *PibH8*, vg0826989447 (C > T, *p* = 4.48 × 10^−13^), is shown with a purple diamond, and the other SNPs are shown in different colors according to their LD (*r* ^2^ value) with the lead SNP. c) Haplotypes of *PibH8* in rice germplasms. *n* denotes the numbers of rice accessions belonging to each haplotype group. Statistical significance was determined by Student's *t*‐test. Seedling survival rate distribution of each haplotype group is shown by box‐plot. d) The expression level of *PibH8* in 18 drought‐sensitive (DS) and 18 drought‐tolerant (DR) associations randomly selected from Hap1 and Hap2 group, respectively. Statistical significance was determined by Student's *t*‐test. e) Transient expression assays of the natural and mutated promoters from two *PibH8* haplotypes. Data represent means ± SD (*n* = 4), and the statistical significance was determined by Student's *t*‐test.

To investigate the genetic basis of drought resistance, we measured seedling survival rate (percentage of survived plants) after severe drought treatment for 237 rice varieties (Table S1, Supporting Information), which were selected from the *indica* subpopulation of a mini‐core germplasm collection. This subpopulation exhibits large genetic variation and a simple population structure compared to the *japonica* subpopulation and the whole population, respectively.^[^ ^25^ ^]^ We then performed GWAS using a mixed linear model (MLM) to identify genetic loci associated with survival rate under drought stress. A major association locus on chromosome 8 (*qRDR8*) was identified (**Figure** **1**a), with the most significant variation, vg0827067928 (*p* = 1.21 × 10^−13^), explaining ≈11% of the phenotypic variance. Within the 200 kb interval (determined by linkage disequilibrium decay value in the population) flanking the leading association signal, 26 non‐transposon coding genes were annotated. RNA‐seq analysis of drought‐stressed leaf samples showed that, among the candidate genes in this interval, an NLR‐encoding gene, *PibH8*, exhibited the most significant change in expression levels after drought treatment (Figure S1, Supporting Information). Additionally, candidate gene association analysis showed that the *PibH8* promoter region were significantly associated with drought‐stressed survival rate (Figure 1b). Thus, *PibH8* was chosen as a candidate gene for further analysis.

The most significant variation in the *PibH8*, vg0826989447 (C > T, *p* = 4.48 × 10^−13^) is located within the promotor region of *PibH8* (Figure 1b). The population was categorized into two primary haplotype groups based on vg0826989447 (frequency of accessions > 0.05; Figure 1c), with *PibH8* ^HapT^ representing haplotype 1 (Hap1, *n* = 126) and *PibH8* ^HapC^ representing haplotype 2 (Hap2, *n* = 111). Hap2 exhibited a significantly (*p* < 0.0001) lower drought‐stressed survival rate compared to Hap1. Therefore, we designated Hap1 as the drought resistance (DR) haplotype and Hap2 as the drought sensitive (DS) haplotype (Figure 1c). Moreover, the expression levels of *PibH8* between DR and DS haplotypes, each represented by 18 accessions randomly selected from Hap1 and Hap2 group, respectively, showed that the DR haplotype (Hap1) had significantly higher *PibH8* expression than the DS haplotype (Hap2) under drought stress condition (*p* < 0.0001) (Figure 1d). Subsequently, the effect of the vg0826989447 variation on the transcriptional activity of the promoter was examined by luciferase activity assay in rice protoplasts. We found that the promoter of *PibH8*‐Hap1 exhibited significantly higher transcriptional activity than the *PibH8*‐Hap2 (Figure 1e), suggesting that SNP vg0826989447 determined the difference in *PibH8* expression levels between the two haplotypes. These results together suggested that *PibH8* is a candidate causal gene at the *qRDR8* locus for drought resistance.

### 2.2 *PibH8* Positively Regulates Drought Resistance in Rice

**Figure 2:**
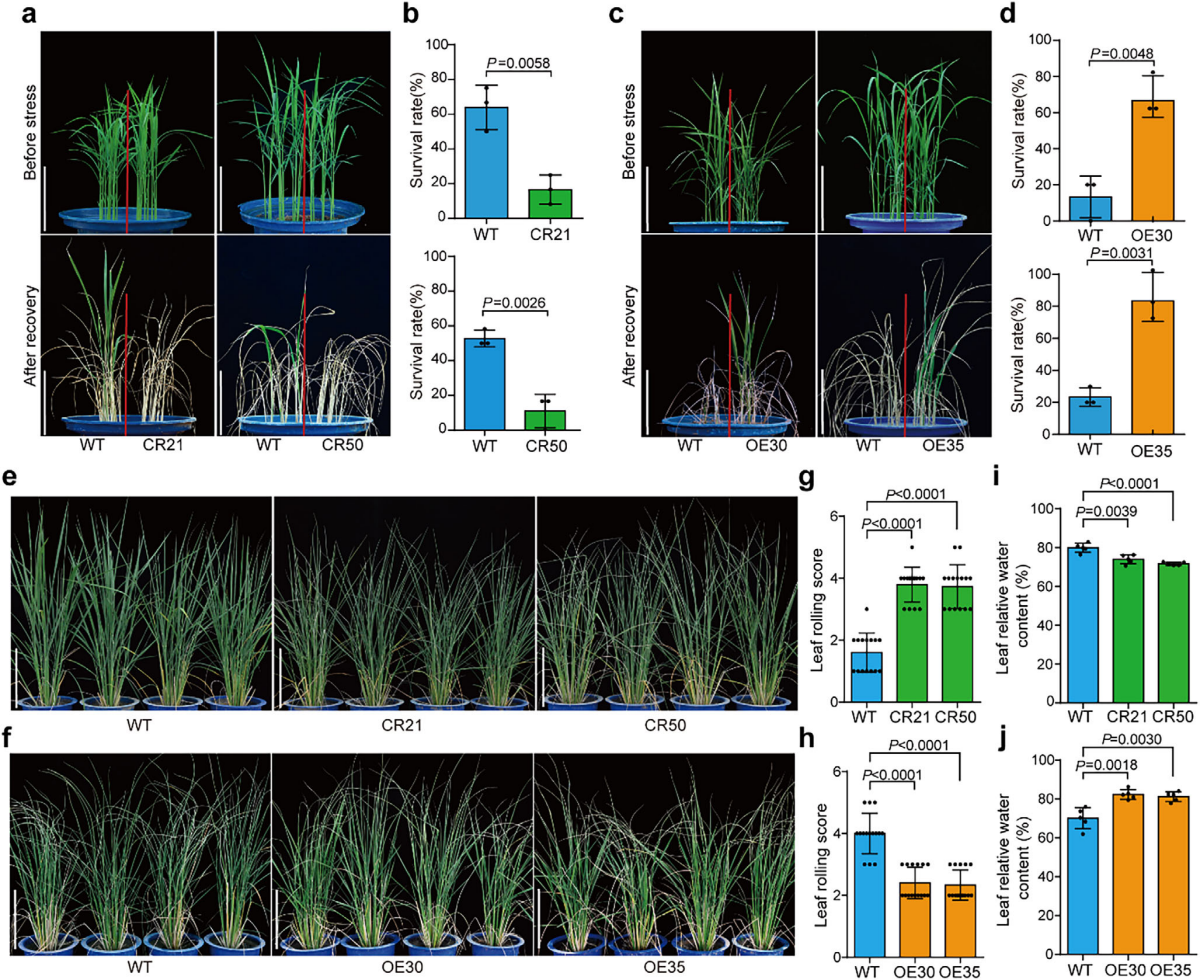
Drought resistance performance of *PibH8* mutant lines and overexpression lines. a,c) Images of *PibH8* mutant lines (a) and overexpression lines (c) before and after recovery from drought stress at the seedling stage. Scale bars, 10 cm. b,d) Survival rates of *PibH8* mutant lines (b) and overexpression lines (d) after drought stress treatment. Data represent means ± SD (*n* = 3). e,f) Images of *PibH8* mutant lines (e) and overexpression lines (f) under drought stress at the panicle development stage. Scale bars, 20 cm. g,h) Comparison analysis of leaf rolling score of *PibH8* mutant lines (g) and overexpression lines (h) under drought stress at panicle development stage. Data represent means ± SD (*n* = 15). i,j) Comparison analysis of leaf relative water content of *PibH8* mutant lines (i) and overexpression lines (j) under drought stress at panicle development stage. Data represent means ± SD (*n* = 5). The significance of all the above data was determined by Student's *t*‐test.

To further confirm *PibH8* as a causal gene in the *qRDR8* controlling drought resistance, we generated *PibH8* knockout mutant lines (CR21 and CR50) using CRISPR‐Cas9 in the Zhonghua11 (ZH11) background (Figure S2a, Supporting Information). Drought resistance testing at the seedling stage revealed that *PibH8* mutant lines exhibited significantly lower survival rates than wild‐type (WT) ZH11 after drought treatment and recovery (**Figure** **2**a,b). We also generated transgenic rice lines overexpressing *PibH8* (OE30 and OE35) (Figure S2b, Supporting Information), which exhibited significantly enhanced drought resistance at the seedling stage (Figure 2c,d). Next, we assessed the drought resistance of the mutant lines and the overexpression lines at the panicle development stage. Under the same level of drought stress, *PibH8* mutant lines exhibited higher leaf rolling scores compared to WT, while the overexpression lines showed much lower leaf rolling scores than WT (Figure 2e–h). By measuring the leaf relative water content (LRWC), we found that the *PibH8* mutant lines had significantly lower LRWC, while overexpression lines had higher LRWC than WT (Figure 2i,j). As *PibH8* mutation has been reported to decrease blast resistance,^[^ ^12^ ^]^ we further tested our *PibH8* mutant and overexpression lines against blast fungus (*Magnaporthe oryzae*) infection. The mutant lines showed significantly increased sensitivity to the infection, consistent with previous reports, while the overexpression lines exhibited significantly increased blast resistance (Figure S3, Supporting Information). Collectively, these results suggest that *PibH8* plays a crucial role in drought resistance, in addition to its known function in blast resistance.

### 2.3 PibH8 Interacts with Phenylalanine Aminotransferases

**Figure 3:**
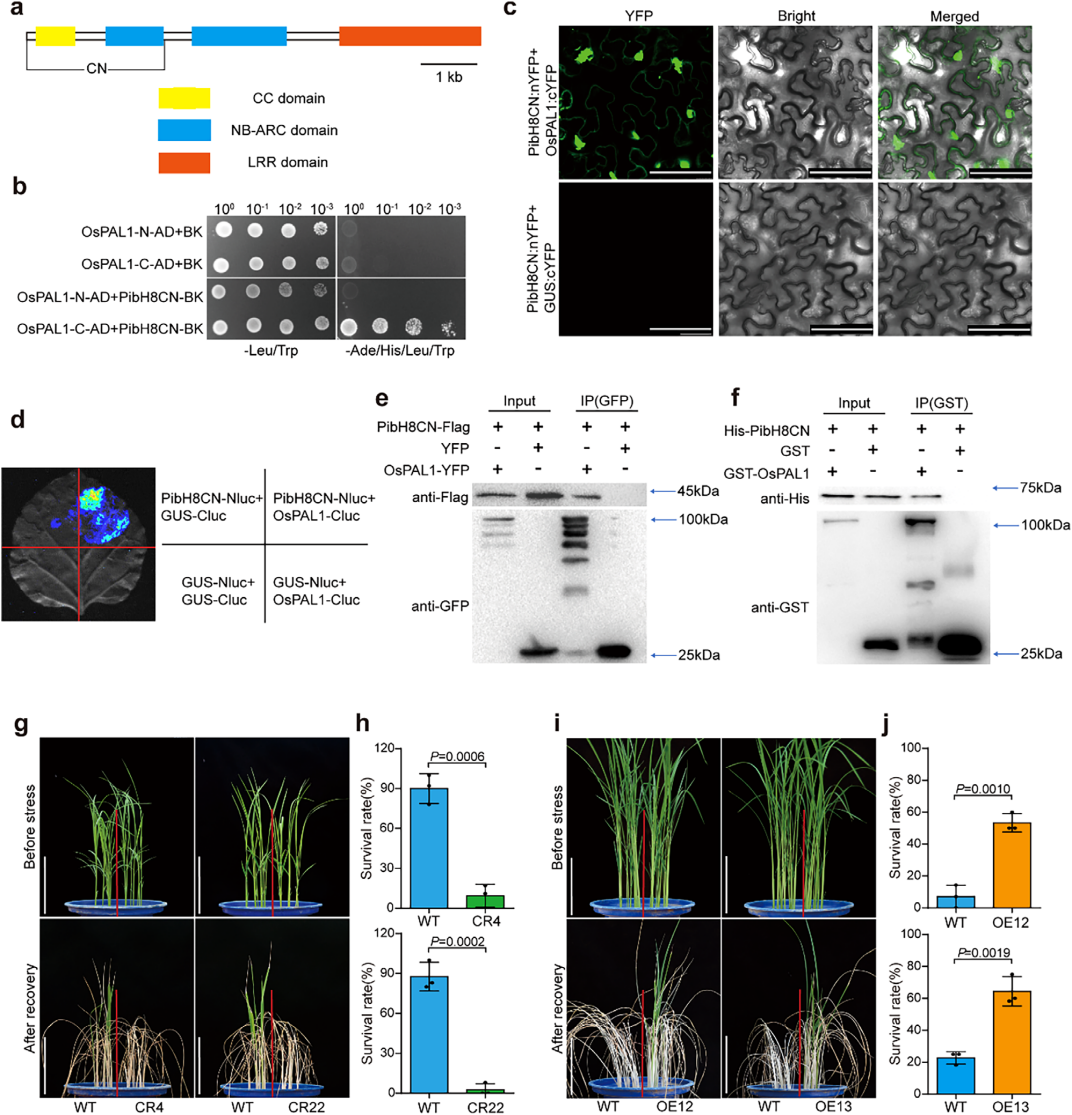
OsPAL1 interacts with PibH8CN and positively regulates drought resistance in rice. a) The NCBI Conserved Domains Search predicted that the PibH8 protein contains four predicted domains, a CC domain, two NB‐ARC domains and a LRR domain. b) Yeast two‐hybrid assay shows PibH8CN can interact with the C‐terminal but not N‐terminal of OsPAL1. c) Bimolecular florescence complementation (BiFC) assay shows the interaction of PibH8CN and OsPAL1 in epidermal cells of *Nicotiana benthamiana* leaves. GUS:cYFP served as a negative control. Scale bars, 100 µm. d) Split firefly luciferase complementation (SFLC) assay shows the interaction of PibH8CN and OsPAL1 in *Nicotiana benthamiana* leaves. GUS‐Cluc served as a negative control. e) Interaction confirmation of PibH8CN and OsPAL1 by Co‐IP in rice protoplasts. Crude protein extracts from expressing 35S:OsPAL1‐YFP and 35S:PibH8CN‐Flag or 35S:YFP and 35S:PibH8CN‐Flag were used as input. Input proteins were immunoprecipitated with GFP‐beads. The input and co‐immunoprecipitated proteins were detected with anti‐GFP and anti‐Flag antibodies as indicated. The approximate positions of the protein molecular weight markers are labeled on the right side. f) Pull‐down assays showed the physical interaction of PibH8CN and OsPAL1. His‐PibH8CN, GST‐OsPAL1, and GST were expressed in *E*. coli strain BL21 (DE3). GST‐OsPAL1 or GST was incubated with His‐PibH8CN and IP with glutathione agarose. Input and pull‐downed proteins were detected with anti‐GST or anti‐His antibodies as indicated. The approximate positions of the protein molecular weight markers are labeled on the right side. g,i) Comparison of *OsPAL1* mutant lines (g) and overexpression lines (i) with WT before and after recovery from drought stress at the seedling stage. Scale bars, 10 cm. h,j) Survival rates of *OsPAL1* mutant lines (h) and overexpression lines (j) compared with WT after drought stress treatment. Data represent means ± SD (*n* = 3). Significance was determined by Student's *t*‐test.

To elucidate the mechanism of PibH8 in regulating the drought resistance, we performed a yeast two‐hybrid screen to identify PibH8‐interacting proteins. Since the CC region of NLRs is often involved in mediating protein‐protein interaction,^[^ ^11^, ^14^, ^15^ ^]^ we used the PibH8CN (1‐363 AA) (**Figure** **3**a) containing the CC and NB‐ARC domains as bait to screen a cDNA library derived from rice seedlings exposed to various abiotic stresses. Several candidate PibH8‐interacting proteins were identified (Table S2, Supporting Information). We focused on OsPAL1 (LOC_Os02g41630) which encodes a phenylalanine aminotransferase that has been reported to involve in regulating multiple stress. We found that PibH8CN could interact with the C‐terminal (395‐701AA) of OsPAL1 but not N‐terminal (1‐394 AA) (Figure 3b). This interaction was further validated using bimolecular fluorescence complementation (BiFC) and split firefly luciferase complementation (SFLC) assays in *Nicotiana benthamiana* (Figure 3c,d). Co‐immunoprecipitation (Co‐IP) assays in rice protoplasts also showed the PibH8CN‐OsPAL1 interaction (Figure 3e). A direct physical interaction between GST‐OsPAL1 and His‐PibH8CN was demonstrated using a pull‐down assay (Figure 3f). These results demonstrate that PibH8CN interacts with OsPAL1. Furthermore, we confirmed the interaction between the full length of PibH8 (PibH8FL) and OsPAL1 using BiFC and SFLC assays in *Nicotiana benthamiana* and Co‐IP assay in rice protoplasts (Figure S4, Supporting Information). Taken together, these results suggest that PibH8 interacts with OsPAL1 likely via its CN domain.

The PAL family have nine members in rice. Since OsPAL9 lacks PAL activity,^[^ ^26^ ^]^ we analyzed the phylogenetic relationship of *OsPAL1‐8* and examined their relative expression levels at the seedling stage (Figure S5, Supporting Information). The results showed that *OsPAL5* is most closely related to *OsPAL1* and exhibits obviously higher expression than other members, except *OsPAL1*. We wondered whether PibH8 could also interact with OsPAL5. Interaction between PibH8 and OsPAL5 were verified using BiFC and SFLC assays in *Nicotiana benthamiana*, Co‐IP assay in rice protoplasts, and pull‐down assay in vitro (Figure S6, Supporting Information). These results suggest PibH8 may interact with multiple PALs in rice, including OsPAL1 and OsPAL5.

### 2.4 *OsPAL1* Positively Regulates Drought Resistance in Rice

*OsPAL1* has been reported to positively regulate rice blast resistance,^[^ ^24^ ^]^ but its role in drought resistance remains unclear. To reveal the function of *OsPAL1* in drought resistance, we generated *OsPAL1* knockout mutant lines (CR4 and CR22) and *OsPAL1* overexpressing transgenic rice (OE12 and OE13) in the ZH11 background (Figure S7, Supporting Information). At the seedling stage, the *OsPAL1* mutants exhibited significantly reduced drought resistance (in terms of survival rate) compared to WT, while the *OsPAL1*‐overexpression lines showed significantly increased drought resistance than WT (Figure 3g–j). Furthermore, the *OsPAL5* mutant line generated by CRISPR‐Cas9 also showed reduced drought resistance (Figure S8, Supporting Information). These results suggest that OsPALs positively affect drought resistance in rice.

### 2.5 PibH8 Interacts with OsFBK16 that Negatively Regulates Drought Resistance

**Figure 4:**
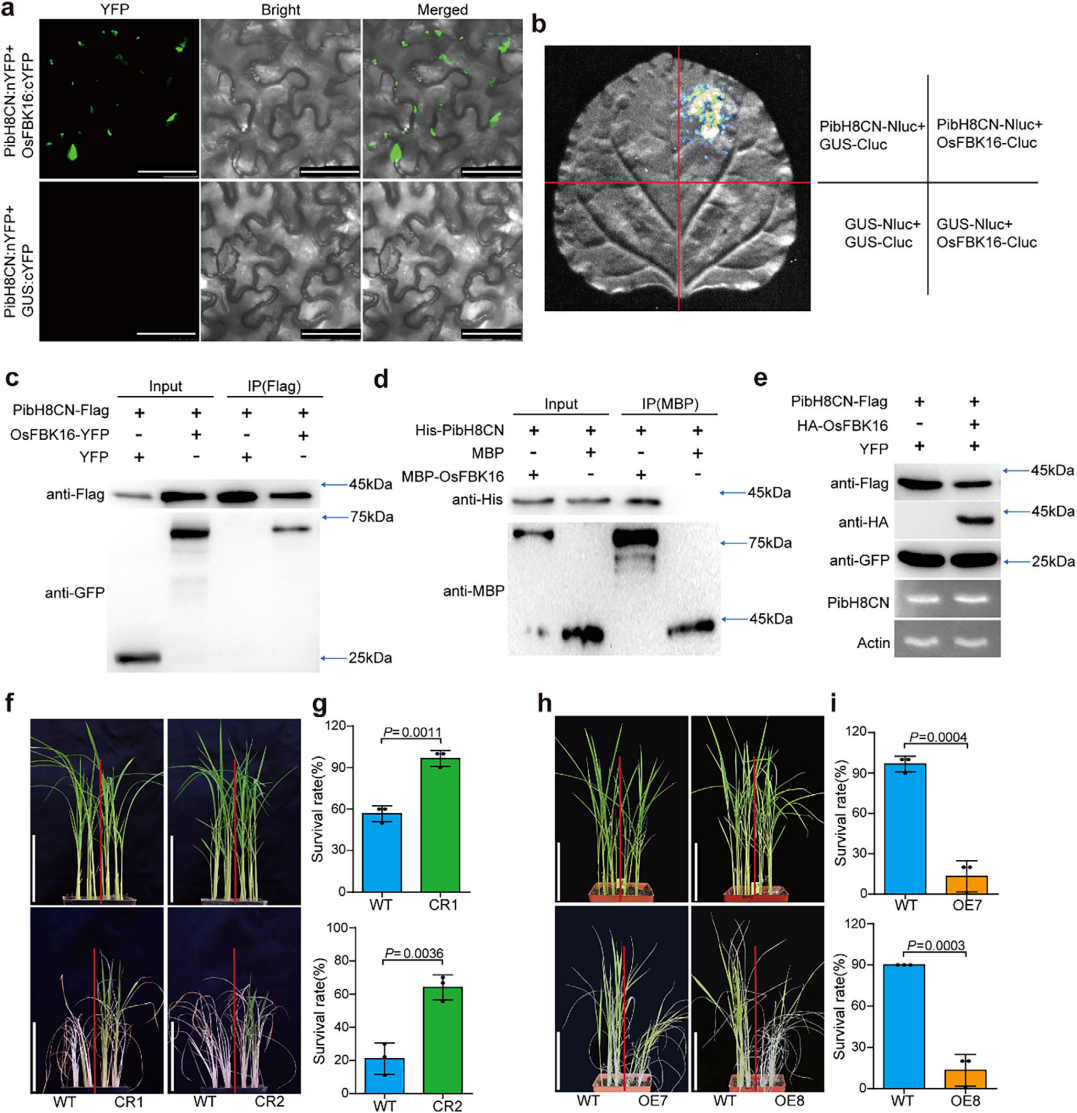
OsFBK16 interacts with PibH8CN and negatively regulates drought resistance. a) BiFC assay shows the interaction of PibH8CN and OsFBK16. GUS:cYFP served as a negative control. Scale bars, 50 µm. b) SFLC assay shows the interaction of PibH8CN and OsFBK16. An unrelated protein GUS served as a negative control. c) Interaction confirmation of PibH8CN and OsFBK16 by Co‐IP in rice protoplasts. 35S:PibH8CN‐Flag was co‐expressed with 35S:OsFBK16‐YFP or 35S:YFP in rice protoplast, followed by total protein extraction. Input proteins were immunoprecipitated with Flag‐beads. The input and co‐immunoprecipitated proteins were detected with anti‐Flag and anti‐GFP antibodies as indicated. The approximate positions of the protein molecular weight markers are labeled on the right side. d) Pull‐down assay shows the interaction of PibH8CN and OsFBK16. Fusion proteins were detected using anti‐His or anti‐MBP antibody. His‐PibH8CN, MBP‐OsFBK16, and MBP were expressed in *E*. coli strain BL21 (DE3). MBP‐OsFBK16 or MBP was incubated with His‐PibH8CN and IP with amylose beads. Input and pull‐downed proteins were detected with anti‐MBP or anti‐His antibodies as indicated. The approximate positions of the protein molecular weight markers are labeled on the right side. e) Co‐transformation assay in rice protoplasts shows that HA‐OsFBK16 can degrade PibH8CN‐Flag. Proteins were detected with Flag, HA, or GFP antibody by WB, and the transcription levels of *PibH8CN* and *actin* were detected by RT‐PCR. f,h) Images of *OsFBK16* mutant lines (f) and overexpression lines (h) with WT before and after recovery from drought stress at the seedling stage. g,i) Survival rates of *OsFBK16* mutant lines (g) and overexpression lines (i) with WT after drought stress treatment. Data represent means ± SD (*n* = 3). Significance was determined by Student's *t*‐test.

OsFBK16, an E3 ubiquitin ligase, has been reported to interact with and degrade PALs in rice, including OsPAL1 and OsPAL5.^[^ ^24^ ^]^ Since OsFBK16, OsPAL1, and PibH8 are all involved in regulating blast resistance in rice^[^ ^12^, ^24^ ^]^ and PibH8 was found to interact with OsPAL1 in this study, we further investigated whether PibH8 is also a substrate of OsFBK16. Based on BiFC and SFLC assays in *Nicotiana benthamiana*, PibH8CN indeed interacted with OsFBK16 (**Figure** **4**a,b), and the interaction was confirmed by Co‐IP assay in rice protoplasts and pull‐down assay in vitro (Figure 4c,d). We further confirmed the interaction of the full‐length PibH8 (PibH8FL) with OsFBK16 by BiFC, SFLC, and Co‐IP assays (Figure S9a–c, Supporting Information). To verify whether OsFBK16 degrades PibH8, we co‐transformed HA‐OsFBK16 with PibH8CN‐Flag/Flag‐PibH8FL in rice protoplasts and determined the PibH8CN/FL protein levels by Western blot (Figure 4e; Figure S9d, Supporting Information). The result showed that the protein levels of PibH8CN and PibH8FL were significantly reduced in the presence of HA‐OsFBK16. We also examined the protein level of PibH8CN in protoplasts from *OsFBK16*‐knockout mutant line (*OsFBK16*‐CR1) and WT. The results showed that PibH8CN protein level was higher in *OsFBK16*‐CR1 protoplasts than in WT protoplasts (Figure S9e, Supporting Information). Furthermore, we conducted a semi‐in vitro ubiquitination assay to test if OsFBK16 ubiquitinated PibH8. The results showed that OsFBK16 ubiquitinates PibH8, and the ubiquitination activity of OsFBK16 was enhanced under drought conditions (Figure S9f, Supporting Information). These results together suggest that OsFBK16 interacts with and degrades PibH8.

OsFBK16 has been reported to negatively regulate blast resistance through degrading OsPALs,^[^ ^24^ ^]^ yet its role in drought resistance remains unknown. The *OsFBK16*‐knockout mutant lines (lines CR1 and CR2) and HA‐OsFBK16‐overexpressing transgenic rice (lines OE7and OE8) in the background of ZH11 (Figure S10, Supporting Information) were tested for drought resistance. At the seedling stage, *OsFBK16* mutant lines had significantly higher survival rates compared to WT (Figure 4f,g), while the overexpression lines showed significantly lower survival rates (Figure 4h,i). Furthermore, we found that the relative enzyme activity of PAL in *OsFBK16*‐OE lines was significantly lower than WT (Figure S11a, Supporting Information). Subsequently, we isolated protoplasts from WT and *OsFBK16*‐OE7 and transfected Flag‐PibH8FL into the protoplasts. With Actin levels maintained consistent as the internal control, we measured the PAL enzyme activities in WT, *OsFBK16*‐OE7, and *OsFBK16*‐OE7 + Flag‐PibH8FL. The results showed that Flag‐PibH8FL could significantly increase the PAL enzyme activity reduced by OsFBK16 (Figure S11b,c, Supporting Information). These results indicate that OsFBK16 negatively regulates drought resistance in rice.

### 2.6 PibH8 Enhances OsPAL1 Stability by Competitively Binding to OsFBK16

**Figure 5:**
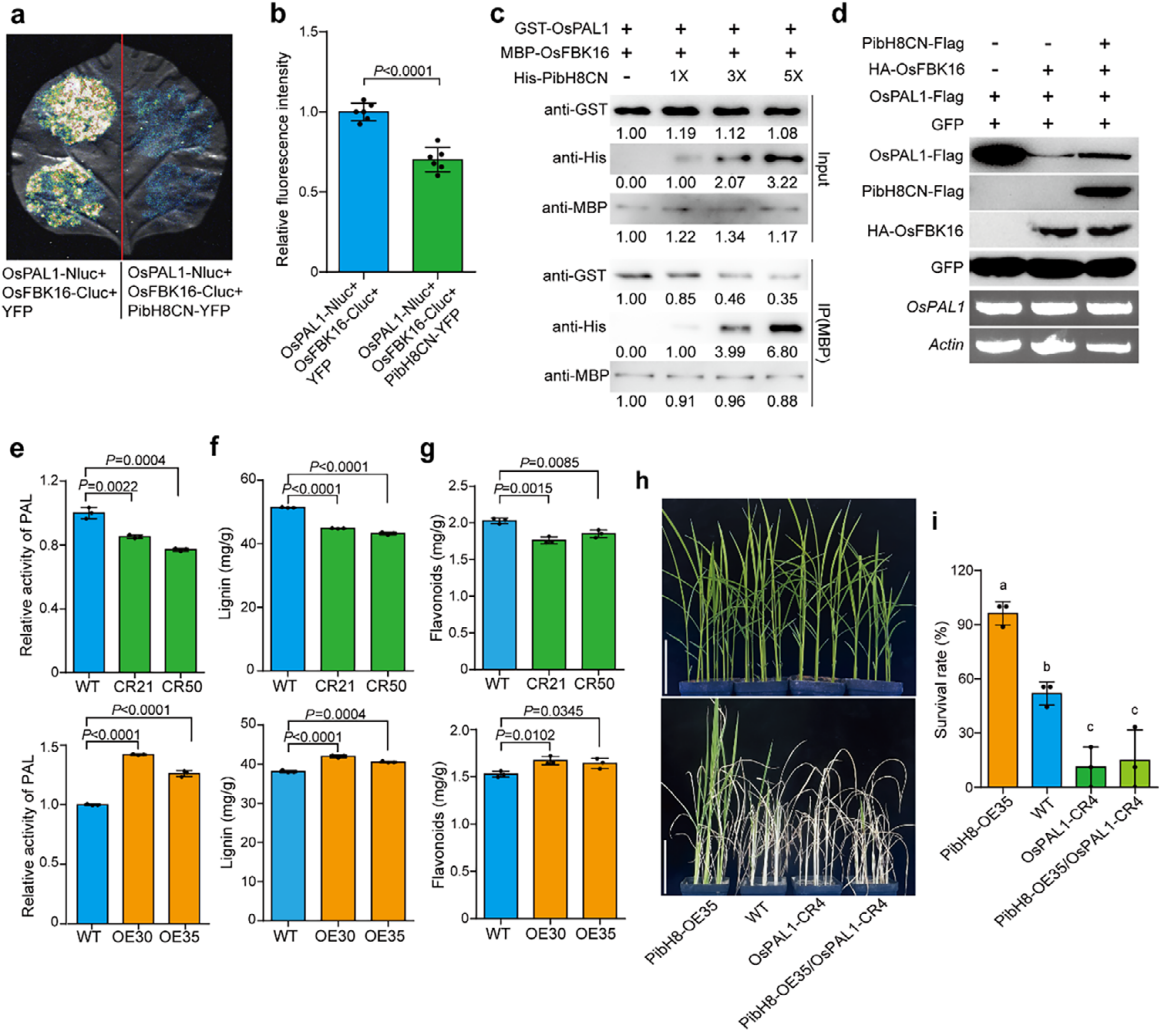
PibH8 enhances the protein stability of OsPAL1 by competitively binding to OsFBK16. a) The competitive SLFC assay in tobacco shows that PibH8CN can inhibit the interaction between OsFBK16 and OsPAL1, and YFP was used as the negative control. b) Statistical analysis of relative fluorescence (LUC) intensity in the competitive SFLC assay. The LUC intensity was normalized using averaged expression levels of LUC and plotted as relative values. Data represent means ± SD (*n* = 6). Significance was determined by Student's *t*‐test. c) The competitive pull‐down assay in vitro shows that PibH8CN can inhibit the interaction between OsFBK16 and OsPAL1, and the interaction intensity between OsFBK16 and OsPAL1 gradually decreases with increasing concentration of PibH8CN. Four equal aliquots of MBP‐OsFBK16 and GST‐OsPAL1 were incubated with amylose beads at 4 °C for 2h, followed by the addition of His‐PibH8CN in corresponding proportions to each aliquot and continued incubation for another 2 h. The input and pull‐downed proteins were detected with anti‐GST, anti‐His, or anti‐MBP antibodies as indicated. The WB bands were quantified and analyzed by Image J. The numerical values are labeled below the bands. d) Co‐transformation assay in rice protoplasts shows PibH8CN inhibits the degradation of OsPAL1 by OsFBK16. Total proteins were extracted from transfected protoplasts and were further detected with Flag, HA, or GFP antibody by WB, and the transcription levels of *Actin* and *OsPAL1* were detected by RT‐PCR. e) Relative activity of PAL in *PibH8* mutant lines and overexpression lines compared to WT. Data represent means ± SD (*n* = 3). Significance was determined by Student's *t*‐test. f) Lignin content of *PibH8* mutant lines and overexpression lines compared to WT. Data represent means ± SD (*n* = 3). Significance was determined by Student's *t*‐test. g) Flavonoids content of *PibH8* mutant lines and overexpression lines compared to WT. Data represent means ± SD (*n* = 3). Significance was determined by Student's *t*‐test. h) Comparison of *PibH8* OE35*, OsPAL1* CR4*, PibH8* OE35*/OsPAL1* CR4 with WT before and after recovery from drought stress at seeding stage. Scale bars, 5 cm. i) Survival rates of *PibH8* OE35*, OsPAL1* CR4, *PibH8* OE35*/OsPAL1* CR4 compared with WT after drought stress treatment. Data represent means ± SD (*n* = 3). Significance was determined by one‐way ANOVA with Tukey's test.

Given that OsFBK16 is known to degrade OsPAL1 and that PibH8 interacts with both OsPAL1 and OsFBK16, we hypothesized that PibH8 may competitively bind to OsFBK16, thereby stabilizing OsPAL1. To prove this, we conducted a binding competition assay based on SFLC in *Nicotiana benthamiana*. We observed that the fluorescence signal intensity of OsFBK16‐OsPAL1 interaction was significantly reduced when PibH8CN‐YFP was added (**Figure** **5**a,b), indicating that PibH8 may interfere the interaction between OsFBK16 and OsPAL1. Next, we performed a binding competition assay based on pull‐down assay in vitro. The result showed that the pulled‐down amount of GST‐OsPAL1 gradually decreased with the gradual increase of added His‐PibH8CN (Figure 5c). Furthermore, we co‐transformed OsPAL1‐Flag and HA‐OsFBK16 in rice protoplasts. Under the premise of consistent transformation efficiency and transcript level, the protein level of OsPAL1‐Flag was significantly decreased. In contrast, the protein level of OsPAL1‐Flag was significantly increased when PibH8CN‐Flag was added (Figure 5d). These results suggest that PibH8CN can enhance the protein stability of OsPAL1 likely through competitively binding to OsFBK16. As the PibH8 could also interact with OsPAL5, we also conducted a competition binding assay based on SFLC in *Nicotiana benthamiana*. The result suggested that PibH8 could also protect OsPAL5 through competition with OsFBK16 (Figure S12, Supporting Information).

To further prove that PibH8 regulates PALs to affect phenylpropane metabolic pathway, we measured the relative PAL activity in the *PibH8* transgenic lines. The results showed that the relative activity of PAL was significantly reduced in the mutant lines and increased in the overexpression lines compared to the WT (Figure 5e). This indicated that PibH8 protects OsPAL1, thereby enhancing PAL activity in rice. We then examined lignin and flavonoids contents in the *PibH8* transgenic lines. The results showed that the lignin and flavonoids contents were reduced in the mutant lines and increased in the overexpression lines compared to WT (Figure 5f,g). Moreover, we detected H_2_O_2_ and MDA contents, as well as the SOD and POD activities, in the *PibH8* transgenic lines. Compared to WT, the H_2_O_2_ and MDA contents were increased in the mutant lines but decreased in the overexpression lines under drought stress. Conversely, the SOD and POD activities were decreased in the mutant lines but increased in the overexpression lines under drought stress (Figure S13, Supporting Information). Furthermore, to examine the genetic relationship between *PibH8* and *OsPAL1*, we generated a transgenic line (*PibH8*‐OE/*ospal1*) with *PibH8* overexpressed in the *ospal1* mutant. After drought stress at seeding stage, the survival rate of *PibH8*‐OE/*ospal1* was similar to that of *ospal1* mutant (Figure 5h,i), indicating that *PibH8* genetically acts upstream of *OsPAL1*. Together, these results suggest that PibH8 protects OsPALs (e.g., OsPAL1 and OsPAL5) through competitively interacting with the E3 ubiquitin ligase OsFBK16, thereby increasing lignin and flavonoids synthesis to improve drought resistance.

### 2.7 The *PibH8*‐Hap.1 is Promising for Drought and Blast Resistance Breeding

Haplotype analysis of *PibH8* showed that the Hap.1 is superior to Hap.2 in conferring drought resistance (Figure 1c). To further confirm this conclusion, we transformed the *PibH8* mutant (CR21) with *PibH8*‐Hap.1 and *PibH8*‐Hap.2, respectively. Drought testing of the complementation lines at the panicle development stage showed that the Hap.1 complementation lines fully rescued the drought‐sensitive phenotype of the *PibH8* mutant, whereas the Hap.2 complementation lines only partially rescued the phenotype (Figure S14, Supporting Information), indicating that the *PibH8*‐Hap.1 is more effective than *PibH8*‐Hap.2 for the genetic improvement of drought resistance in rice.

**Figure 6:**
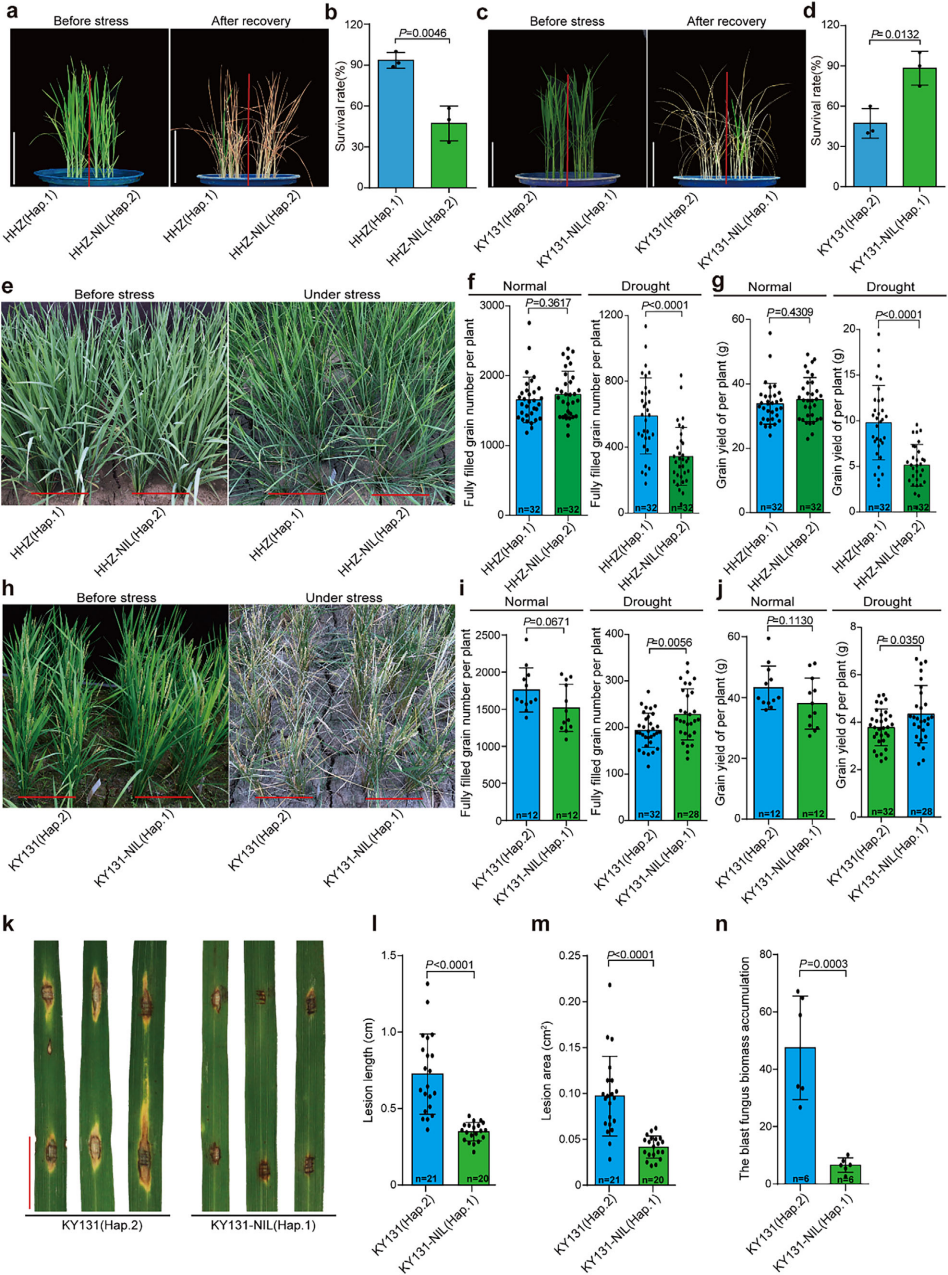
Drought and blast resistance performance of *PibH8* NILs. a) Images of HHZ (Hap.1) and HHZ‐NIL (Hap.2) before and after recovery from drought stress at the seedling stage. b) Survival rates of HHZ (Hap.1) and HHZ‐NIL (Hap.2) after drought stress treatment. Data represent means ± SD (*n* = 3). c) Images of KY131 (Hap.2) and KY131‐NIL (Hap.1) before and after recovery from drought stress at the seedling stage. d) Survival rates of KY131 (Hap.2) and KY131‐NIL (Hap.1) after drought stress treatment. Data represent means ± SD (*n* = 3). e) Images of HHZ (Hap.1) and HHZ‐NIL (Hap.2) under drought stress in the field. f) Fully filled grain number per plant of HHZ (Hap.1) and HHZ‐NIL (Hap.2) under normal and drought conditions in the field. Data represent means ± SD (*n* ≥ 30). g) Grain yield of per plant of HHZ (Hap.1) and HHZ‐NIL (Hap.2) under normal and drought conditions in the field. Data represent means ± SD (*n* ≥ 30). h) Images of KY131 (Hap.2) and KY131‐NIL (Hap.1) under drought stress in the field. i) Fully filled grain number per plant of KY131 (Hap.2) and KY131‐NIL (Hap.1) under normal and drought conditions in the field. Data represent means ± SD (*n* ≥ 12). j) Grain yield of per plant of KY131 (Hap.2) and KY131‐NIL (Hap.1) under normal and drought conditions in the field. Data represent means ± SD (*n* ≥ 12). k) Images of KY131 (Hap.2) and KY131‐NIL (Hap.1) after inoculation with blast fungus (*Magnaporthe oryzae*, isolate *TLP37*). Scale bars, 1cm. l) The lesion length of KY131 (Hap.2) and KY131‐NIL (Hap.1) after inoculation with blast fungus. Data represent means ± SD (*n* ≥ 20). m) The lesion area of KY131 (Hap.2) and KY131‐NIL (Hap.1) after inoculation with blast fungus. Data represent means ± SD (*n* ≥ 20). n) The blast fungus biomass accumulation on KY131 (Hap.2) and KY131‐NIL (Hap.1) leaves after inoculation. Data represent means ± SD (*n* = 6). The significance of all the above data was determined by Student's *t*‐test.

As *PibH8* has been recently reported as a homolog of blast resistance gene *Pib*,^[^ ^12^ ^]^ we further investigated whether the *PibH8*‐Hap.1 holds greater value than *PibH8*‐Hap.2 in rice breeding for both drought and blast resistance by generating two near‐isogenic lines (NILs) for evaluation. One NIL was constructed by replacing the *PibH8*‐Hap.1 allele in the elite *indica* variety Huanghuazhan (HHZ) with the *PibH8*‐Hap.2 allele from the drought‐sensitive rice WAB462 (W119). Another NIL was created in the elite *japonica* variety Kongyu131 (KY131), in which the *PibH8*‐Hap.2 allele was replaced with the *PibH8*‐Hap.1 from HHZ. As expected, the expression level of *PibH8* in HHZ‐NIL was significantly lower than that in HHZ under drought stress, but no difference under the normal growth conditions (Figure S15a, Supporting Information). Conversely, KY131‐NIL showed significantly higher *PibH8* expression than KY131 under drought stress, with no difference under the normal growth conditions (Figure S15b, Supporting Information). Furthermore, we examined the lignin and flavonoids accumulation in the HHZ‐NIL. The lignin and flavonoids contents in HHZ‐NIL were significantly lower than those in HHZ under drought stress; however, there was no difference between the two materials under normal growth conditions (Figure S15c,d, Supporting Information). Drought testing at seedling stage showed the survival rate of HHZ‐NIL was significantly lower than that of HHZ, while the survival rate of KY131‐NIL was significantly higher compared to KY131 (**Figure** **6**a–d). Under drought stress in the field, the HHZ‐NIL plants showed much early leaf‐rolling at panicle development stage compared to HHZ, whereas the KY131‐NIL plants showed much delayed leaf‐rolling in contrast to KY131 (Figure 6e,h). After recovery, HHZ‐NIL plants showed a significantly decrease in the number of fully filled grain and yield per plant compared to HHZ (Figure 6f,g). Conversely, KY131‐NIL plants showed significantly higher number of fully filled grain and yield per plant than KY131 (Figure 6i,j). Under well‐irrigated conditions, the NILs plants had similar performance in the number of fully filled grain and yield per plant compared to their respective control plants (Figure 6f,g,i,j). Moreover, there were no significant difference in other agronomic traits between the NILs and their control plants (Figure S16, Supporting Information). We further tested the blast resistance of the KY131‐NIL growing in the field. After inoculated with blast fungus (two isolates tested), the KY131‐NIL leaves exhibited significantly increased blast resistance, indicated by the lesion length and area and blast fungus biomass accumulation, compared to KY131 (Figure 6k–n; Figure S17, Supporting Information). Collectively, these results suggest that the *PibH8*‐Hap.1 allele holds promising breeding value for improving both drought and blast resistance in rice.

**Figure 7:**
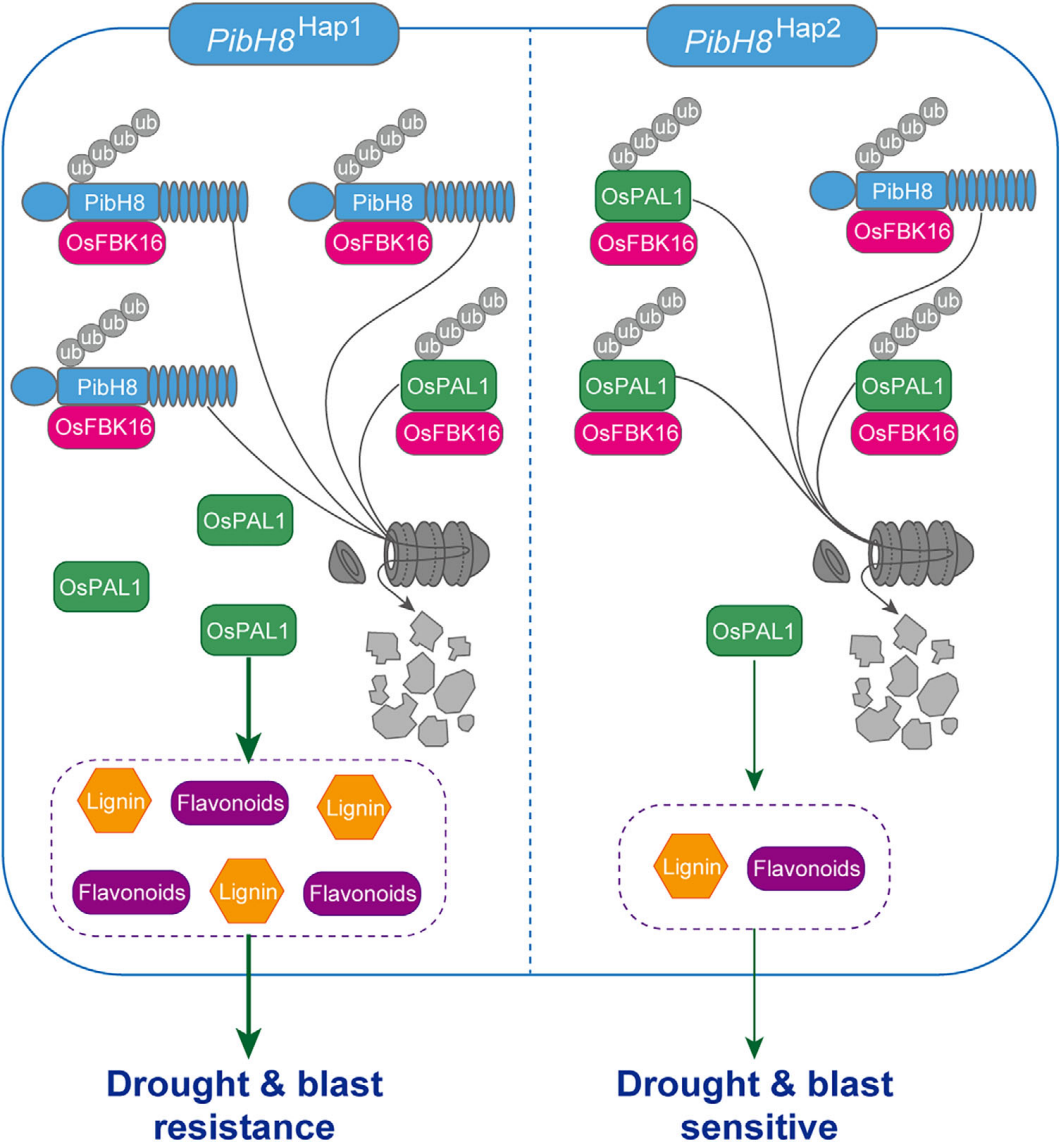
A proposed working model for *PibH8* in regulating drought and blast resistance. The Hap1 allele of *PibH8* has a higher expression level and the accumulation of more PibH8 proteins protects more OsPAL1 protein, which results in higher drought and blast resistance in Hap1 accessions (left) than in Hap2 accessions (right).

In conclusion, we characterized an NLR protein, PibH8, that positively regulates drought resistance by competitively binding to an E3 ubiquitin ligase OsFBK16 to protect a phenylalanine aminotransferase OsPAL1, leading to enhanced accumulation of the lignin and flavonoids. The causal variation in the *PibH8* promoter resulted in two *PibH8* haplotypes with differential expression. The *PibH8*‐Hap1 with a higher expression level can protect more OsPAL1 protein for synthesis of lignin and flavonoids, resulting in stronger drought and blast resistance than *PibH8*‐Hap2 (**Figure** **7**). Our findings suggest that *PibH8*‐Hap1 is a valuable breeding target for improving both drought and blast resistance.

## 3 Discussion

Previous studies have shown that NLRs are mainly involved in the activation of effector‐triggered immunity processes in the organism, thereby conferring disease resistance to plants.^[^ ^5^, ^27^ ^]^ To date, the role of NLR genes in the regulation of abiotic stress, particularly in drought resistance, has been poorly understood. In this study, we identified a blast resistance gene *PibH8* by GWAS, which plays a positive regulatory role in drought resistance at both seedling and panicle development stages (Figure 2). As a typical CNL‐type NLR gene, *PibH8* has been reported to be a homolog of *Pib* and its mutant showed blast‐sensitive phenotype.^[^ ^12^ ^]^ Our results showed the lesion lengths of *PibH8* mutant lines were longer and the lesion areas were bigger compared with the wild‐type, whereas the overexpression lines of *PibH8* had the opposite phenotype (Figure S3, Supporting Information). These results indicate that *PibH8* positively regulates both drought and blast resistance. There are a few reports showing that some transcription factors such as OsNAC6 and OsWRKY45 regulate both blast and drought resistance,^[^ ^28^ ^]^ however, to the best of our knowledge, no NLR protein has been reported to confer both drought and blast resistance. Therefore, our findings significantly expand the functional diversification of NLRs in plant‐environment interaction.

PAL has been characterized as rate‐limiting enzyme in the phenylpropane metabolic pathway. Its function encompasses a diverse range of environmental stimuli, including pathogen infection, wounding, ultraviolet irradiation, and various other stress conditions.^[^ ^24^, ^29^ ^]^ In this study, we found that PibH8 interacts with OsPAL1 (Figure 3b–f; Figure S4, Supporting Information). A previous study showed that an E3 ubiquitin ligase OsFBK16 interacts with OsPAL1 and promotes its degradation, thus negatively regulating rice blast resistance.^[^ ^24^ ^]^ Here, we found that OsFBK16 also interacts with PibH8 and negatively regulates drought resistance (Figure 4; Figure S9, Supporting Information). It has been reported that NLR proteins regulate the interaction between other two proteins through competition, thus playing a protective role. For example, the NLR protein PigmR can interfere the AVRpi9‐PICI1 interaction to inhibit the degradation of PICI1 by AVRpi9, thereby protecting PICI1 to positively regulate rice blast resistance.^[^ ^15^ ^]^ In addition, the NLR protein SW‐5B activates antiviral immunity by binding to PP2C4, thereby deregulating the inhibition of SnRK2.3/2.4 by PP2C4.^[^ ^30^ ^]^ Based on our results, the competitive mechanism of PibH8 seems similar to that of PigmR and SW‐5B (Figure 5a–d). We propose that some NLR proteins (like PibH8, PigmR, and SW‐5B) act as a ‘guarder’ by binding to a ‘repressor’ (like OsFBK16, AVRpi9, and SnRK2.3/2.4) and then releasing the repressor from inhibition of the downstream proteins (like OsPAL1, PICI1, and PP2C4), thus playing an essential role in protecting downstream proteins. This mechanism may provide a valuable clue for functional characterization of a large number of other NLR proteins.

Lignin and flavonoids are important secondary metabolites of the phenylpropanoid pathway that have been reported to be involved in the regulation of a variety of stresses.^[^ ^31^ ^]^ For example, OsMYB30 has been reported to increase lignin content, thicken thick‐walled tissue cells, and inhibit rice blast infection by activating the expression of *Os4CL3* and *Os4CL5*, thereby improving rice blast resistance.^[^ ^32^ ^]^ Whereas overexpression of a tea flavanone 3‑hydroxylase gene, *CsF3H*, confers tolerance to salt and *Alternaria solani* in transgenic tobacco.^[^ ^33^ ^]^ Previous studies have shown that OsPAL1 acts as the first rate‐limiting enzyme in the phenylpropanoid pathway and that expression of the *OsPAL1* gene is required for the biosynthesis of secondary metabolites, including lignin and SA.^[^ ^22^, ^34^ ^]^ In this study, we found that the PAL family member OsPAL1 also positively regulates drought resistance in rice (Figure 3g–j). As PibH8 competitively interacts with OsFBK16 to protect OsPAL1 (Figure 5a–d), we found that the relative activity of PAL and lignin and flavonoids content were decreased in the *PibH8* mutant lines, whereas they were increased in the *PibH8* overexpression lines (Figure 5e–g). This suggests that PibH8 may promote the accumulation of lignin and flavonoids by competitively protecting the PAL family members such as OsPAL1, thereby enhancing drought and blast resistance.

Climate change frequently causes sequential or simultaneous occurrence of abiotic stresses and diseases in the major rice‐growing areas. For instance, the alternation of rainy and dry seasons may be irregular in some areas, and there may be prolonged periods of drought stress, inhibiting rice growth and reducing its physiological functions. After drought stress, sudden rains with increased air humidity allow the rice to resume growth rapidly, but at the same time provide a favorable environment for the rice blast fungus infection.^[^ ^35^ ^]^ To date, many rice blast resistance genes, such as *Pib*, *PigmR*, and *Pijx*,^[^ ^10^, ^13^, ^36^ ^]^ and drought resistance genes, such as *DROT1* and *DRG9* ^[^ ^4^, ^37^ ^]^ have been cloned, and some of them have been successfully applied in rice breeding. However, very few genes have been reported with breeding value for improving both drought and blast resistance. In this study, we found that *PibH8* has two major haplotypes, and the two haplotypes have differences in expression and drought resistance, which are mainly caused by the mutation of SNP9447 in the promoter region (Figure 1c–e). Interestingly, the blast and drought resistance in KY131, a variety that has been widely planted in northeastern China, were significantly improved after introgression with the superior allele *PibH8* ^Hap.1^ (Figure 6c,d,h,j). Notably, there were no significant difference in the important agronomic traits between the NILs and their recipient variety under normal growth conditions (Figure S16, Supporting Information). All these results indicate that the *PibH8* ^Hap.1^ haplotype has promising value for drought and blast resistance breeding. In facing the challenges of food security and sustainable agriculture, developing crops resilient to multiple stresses, such as drought and blast disease in rice, becomes increasingly important and urgent. Exploring superior alleles of multi‐stress‐resistance genes, either by mining the natural variations in germplasms or by gene editing, are practically effective strategies for genetic improvement of elite high‐yielding varieties with inferior alleles for multi‐stress resilience. For example, RiceNavi is a highly useful platform that integrates rice genomic, phenotypic, and breeding data.^[^ ^38^ ^]^ Variation information for *PibH8* and its environmental interaction effects (e.g., expression regulation patterns under drought conditions) can be input into the database to establish a “genotype‐phenotype‐environment” association network, guiding parental line selection and multi‐gene pyramiding designation. Precise selection of *PibH8* ^Hap.1^ combined with other superior drought‐ and/or blast‐resistant alleles, may be a solution to breed drought‐ and blast‐resistant varieties for regions prone to drought and blast disease.

In summary, we found a NLR gene, *PibH8*, that positively regulates drought and blast resistance in rice. This study not only elucidates an unreported mechanism by which NLR proteins modulate stress resistance through competitively interaction with PALs but also highlights the *PibH8* gene as a promising candidate for developing rice varieties with drought and blast resistance.

## 4 Experimental Section

### Drought Resistance Phenotyping of the Rice Population and GWAS

Drought resistance phenotyping was performed on a natural population consisting of 237 *indica* accessions (Table S1, Supporting Information) selected from a core panel of rice germplasms.^[^ ^25^ ^]^ The phenotyping experiment was conducted at the drought resistance testing station (30°28′15.84″ N, 114°21′6.42″ E) of Huazhong Agricultural University in Wuhan, China. To implement drought treatment, the rice field (55 m × 15 m) was equipped with a movable rain‐off shelter, which could be deployed to prevent rainfall and maintain drought stress during rainy days. The experimental soil was formulated as a 1:2 volumetric mixture of river sand and field soil. The plots were surrounded by 2.0‐m‐deep reinforced concrete perimeter trenches featuring evenly distributed perforations along the inner walls. Flood irrigation was implemented to maintain a surface water depth of 1–2 cm during normal watering phases. Drought stress was applied at the three‐leaf stage of rice seedlings by halting irrigation, draining water from the field, and closing the shelter during rainfall to allow the field to dry gradually. The rice field was subjected to 30 days of drought stress at the seedling stage. Throughout the drought stress period, the soil moisture at 25 cm depth was monitored using frequency domain reflectometry (FDR), and the air temperature and humidity, ranging from 18 to 43 °C and 23–100%, respectively, were also recorded. The drought treatment was ceased when 70% of the plants in the population exhibited irreversible leaf rolling in the morning, at which point the soil water content had decreased to 14.3%. Following drought stress, the field was irrigated for 7 days to facilitate rice recovery. The 237 accessions, with three replications (711 plots), were planted in the field following a completely randomized block layout. Each plot (≈18 cm × 18 cm) contained 25 rice plants. Survival rates (percentage of survived plants each plot) were measured after drought recovery.

GWAS was performed using the high‐density SNP dataset from RiceVarMap v.2.0.^[^ ^25^ ^]^ High‐quality genotype data for 7 367 740 genomic variations (with a minor allele frequency ≥ 0.05 and at least six accessions carrying the minor allele) covering the entire rice genome were used to conduct GWAS using a linear mixed model. The model accounted for kinship, estimated from genetic similarities, as a random effect, with data provided by the FaST‐LMM program.^[^ ^39^ ^]^ The kinship (K) matrix for all accessions was calculated using PLINK.^[^ ^40^ ^]^ The genome‐wide suggestive (*p* = 6.54 × 10^−7^) and significant (*p* = 3.27 × 10^−8^) thresholds for the GWAS were calculated using the GEC software tool,^[^ ^41^ ^]^ to control the genome‐wide type I error rate. Potential candidate genes within the 200‐kb interval centered on the lead variation from the GWAS,^[^ ^42^ ^]^ and the variations (*p*‐value < 3.27 × 10^−8^) were determined based on the local LD level using the “–clump” command of Plink.^[^ ^40^ ^]^ Visualization of candidate gene‐associated mapping based on the GWAS results and pairwise LD analysis was performed by LDBlockShow.^[^ ^43^ ^]^

### Vector Constructions and Genetic Transformation

To generate *PibH8*, *OsPAL1*, and *OsFBK16*‐OE lines, the full‐length coding sequences of *PibH8, OsPAL1*, and *OsFBK16* were amplified from rice Nipponbare using primers PibH8/OsPAL1/OsFBK16‐OE‐F/R (Table S3, Supporting Information). The amplified product of *PibH8* was then introduced into the binary vector pCAMBIA1301U (digested by *Kpn*I and *Bam*HI) using Gibson assembly.^[^ ^44^ ^]^ And the amplified product of *OsPAL1/OsFBK16* were introduced into the binary vector pCAMBIA1301U‐Flag/HA with minor modification. For the loss‐of‐function mutants, two sites in the coding region of *PibH8* were targeted, and two small‐guide RNA sequences were cloned into pRGEB32 vector to generate the *PibH8* CRISPR‐Cas9 construct, following the method as described previously.^[^ ^45^ ^]^ The loss‐of‐function mutants of *OsPAL1* and *OsFBK16* were generated by using the TKC CRISPR‐Cas9 vector.^[^ ^46^ ^]^ All the sequence‐confirmed constructs were introduced into ZH11 by following the standard protocol for *Agrobacterium tumefaciens*‐mediated transformation.^[^ ^47^ ^]^ Primers used for vector construction are listed in Table S3 (Supporting Information).

### Drought Resistance Evaluation of Transgenic and Near‐Isogenic Lines (NILs)

For drought testing at the seedling stage, rice seeds were geminated on 1/2 MS medium. At 5 days after germination, the seedlings were transplanted into pots filled with water‐saturated soil. Each pot contained two genotypes (i.e., mutant line and WT) with an equal number (*n* = 10–12 plants per genotype) of plants for phenotypic observation, and at least three repeats were performed. Plants at the four‐leaf stage were stressed with drought by stopping watering for 7–10 d, and re‐watered until significant differences in seedling wilting were observed. After recovery, plants with green leaves and regenerating shoots were considered as survived plants and the survival rate (percentage of survived plants) was recorded.

For drought stress testing at the panicle development stage, plants were planted in pots for drought treatment and trait measurement. When rice plants grew to the panicle development stage, irrigation was then stopped to impose drought stress. The soil water content was monitored by weighing. When the soil water content decreased to 10%, the plants were watered once per day to maintain the soil water content at 10% for about ≈7 days. When difference in leaf rolling appeared, the leaf rolling score was recorded and the leaf relative water content was measured. For drought stress testing in field, NILs and their control materials were planted in the fields which assessed for drought resistance in Wuhan. When rice plants grew to the panicle development stage, irrigation was then stopped to impose drought stress. The rice pants were re‐watering after ≈15 days and subsequently collect yield traits. The filed drought stress treatment period spanned approximately from July to August. During this period, the average daytime maximum temperature fluctuated between 30 and 35 °C, occasionally reaching to 39 °C. At night, the temperature generally ranged from 24 °C to 29 °C. The relative humidity was maintained between 60% and 80%. The number of replicates for all the statistical analyses is provided in the figures, and the significance of the data was determined by Student's *t*‐test.

The leaf rolling score was slightly adjusted according to the five‐grade scoring method of O'Toole et al. (1980).^[^ ^48^ ^]^ Grade 1: no symptoms of leaf rolling, or below 20% leaf rolling; Grade 2: 20–40% leaf rolling; Grade 3:41–60% leaf rolling; Grade 4:61–80% leaf rolling; Grade 5: more than 81% leaf rolling. The leaf relative water content (LRWC) was calculated by formula LRWC = (FW‐DW)/(SW‐DW) ×100%, in which fresh weight (FW) of the leaves was weighed immediately after sampling, saturated weight (SW) was weighed at 24 h after being immersed in distilled water in the dark, and dry weight (DW) of the leaves was weighed after being baked in an oven at 80 °C for more than 48 h.

### Construction and Field Testing of NILs

Two NILs were constructed by recurrent backcrossing. One is Huanghuazhan (HHZ)‐NIL(Hap.2), which contains a short segment harboring *PibH8*‐Hap.2 from W119, a *japonica* germplasm harboring *PibH8*‐Hap.2, in the genetic background of HHZ, an elite indica variety harboring *PibH8*‐Hap.1. Another NIL was constructed in the genetic background of Kongyu131(KY131), an elite *japonica* variety harboring *PibH8*‐Hap.2, by introducing the *PibH8*‐Hap.1 from HHZ. Marker Id8R2700 was used to select target lines containing the *PibH8* different allele from the BC_1_F_1_ to the BC_4_F_3_ generations (Table S3, Supporting Information). HHZ‐NIL (Hap.2) carries a 260‐kb genomic segment (between markers Ind8X2686 and Ind8X2713) from W119 containing *PibH8*‐Hap.2, while KY131‐NIL(Hap.1) carries a 600‐kb genomic segment (between markers Ind8X2686 and Ind8X2742) from HHZ containing *PibH8*‐Hap.1. Primers for the markers are listed in Table S3 (Supporting Information). The NILs and their control rice were planted in a drought‐stressed field plot at Wuhan to evaluate drought resistance. HHZ‐NIL (Hap.2) and its control rice were grown at Wuhan and examined for agronomic traits under normal irrigation condition. KY131‐NIL (Hap.1) and its control rice were grown in Jiansanjiang, where the KY131 variety had been dominating in the rice production, and examined for agronomic traits under normal condition.

### Blast Resistance Evaluation

For seedling inoculation, the leaves of 3–4 leaf stage were punch‐inoculated using as previously described.^[^ ^14^ ^]^ Specifically, spores were collected in sterile water from each isolate grown on complete agar medium for 10 days at 25 °C under a 12‐h light/12‐h dark photoperiod. The blast fungus spore (*TLP37* or *YC6* isolates) suspensions were adjusted to a density of ≈1 × 10^5^ spores m^−1^. The leaves were treated in the dark at room temperature for 1 day, and then cultured in a light culture room at 28 °C with ≈90% humidity. Disease symptoms were evaluated 7 days post‐inoculation by measuring the lesion area and length on over 15 infected leaves using ImageJ software. The blast fungus biomass accumulation was quantified by quantitative PCR with primers listed in Table S3 (Supporting Information). The significance of the above data was determined by Student's *t*‐test.

### RNA Isolation and Quantitative PCR

Total RNAs from rice organs including leaf and axillary bud were extracted using the TRIzol reagent (Invitrogen) and then 2 µg total RNA was reversely transcribed using EasyScript® One‐Step gDNA Removal and cDNA Synthesis SuperMix (AE311; Transgen, Beijing, China) according to the manufacturer's instructions. Quantitative reverse transcription polymerase chain reaction (qRT‐PCR) was performed with PowerUp^TM^ Applied Biosystems^TM^ SYBR^TM^ Green Master Mix (A25742; Foster City, CA, USA) on an RT‐PCR system (StepOnePlus or QuantStudio 6 Flex; Applied Biosystems), with the rice *Ubiquitin* gene (LOC_Os03g13170) used as an internal control. The primers of the genes detected in qRT‐PCR experiments are listed in Table S3 (Supporting Information).

### Luciferase Activity Assay in Rice Protoplasts

To investigate the effect of the *PibH8* promoter variations on the gene expression, transient assays were conducted using protoplasts from rice seedling. Promoter fragments of *PibH8* amplified from two representative varieties, HHZ (Hap.1) and W119 (Hap.2), as well as three modified promoters, were amplified and inserted into the pGreenII 0800‐LUC vector^[^ ^49^ ^]^ at the *Bam*HI and *Kpn*I digestion sites. The resulting constructs, with 2‐3 µg of each, were transfected into rice protoplasts. The isolation and transformation of rice protoplasts following the method previously described.^[^ ^50^ ^]^ Luciferase activity was measured using the Dual Luciferase Reporter Assay System (Promega) according to the manufacturer's instructions.

### Recombinant Protein Expression and Purification

Recombinant His‐PibH8CN was inserted at the *Eco*RI /*Sal*I sites of pET32a, and GST‐OsPAL1 was inserted at *Bam*HI/*Sal*I sites of PGEX‐4T‐1. OsFBK16 was cloned into pMAL‐C2X at the *Eco*RI/*Sal*I sites to express recombinant MBP‐OsFBK16. These plasmids were transformed into *E. coli* BL21 (DE3) cells. Recombinant proteins were induced overnight at 16–18 °C by the addition of 1.0 mm isopropylthio‐β‐galactoside when cell density at OD_600_ reached 0.6. The proteins were purified using gravity flow with Ni‐NTA beads (Thermo) for His‐tagged proteins, glutathione beads (GE Healthcare) for GST‐tagged, and amylose beads (NEB) for MBP‐tagged proteins.

### Pull‐Down Assays

The recombinant GST‐fused OsPAL1 was purified from bacteria using glutathione beads (GE Healthcare), while His‐PibH8CN was purified using Ni‐NTA beads (Thermo) and MBP‐OsFBK16 was purified using amylose beads (NEB). 1 µg of His‐PibH8CN were incubated with 1 µg GST‐OsPAL1/GST using Glutathione beads or 1 µg MBP‐OsFBK16/MBP using amylose beads as indicated in pull‐down buffer (20 mm Tris‐HCl, pH 7.5, 100 mm NaCl, 1 mM EDTA) at 4 °C for 4 h. The beads were then washed five times with wash buffer (20 mm Tris‐HCl, pH 7.5, 100 mm NaCl, 1 mM EDTA, 0.5% Nonidet P‐40,). The proteins were eluted from beads by boiling in 100 µl 2×SDS sample buffer andseparated on 10% SDS‐PAGE gels. Gel blots were detected by anti‐His (ABclonal, AE003, 1:5000 dilution), anti‐GST (ABclonal, AE001, 1:5000 dilution), and anti‐MBP (ABclonal, AE016, 1:5000 dilution) antibodies.

### Split Firefly Luciferase Complementation Assays

Split firefly luciferase complementation assays were performed as described previously.^[^ ^51^ ^]^ The sequences of PibH8CN/FL, OsPAL1, and OsFBK16 were cloned into the vectors pJW771 and pJW772 at *Bam*HI and *Sal*I digestion sites, respectively. All constructs were transformed into Agrobacterium GV3101 and then injected into tobacco (*N. benthamiana*) leaves. After 48 h, 1 mm luciferin (LUCK‐1G; GOLDBIO) was applied uniformly to the surface of the leaves and the images were captured in the dark using a Lumazone imaging system, equipped with a 2048B CCD camera (Roper).

### BiFC Assays

*PibH8CN/FL*, *OsPAL1*, and Os*FBK16* sequences were amplified by PCR and cloned into the pDONR223 vector using the BP recombination reaction (Invitrogen). LR reactions were performed with the target vector pEarlyGate‐35S‐n/cYFP and various pDONR223 vectors. Agrobacterial suspensions containing p35S: PibH8CN/FL:nYFP and p35S:OsPAL1/OsFBK16:cYFP constructs were injected into abaxial epidermis of tobacco leaves. The fluorescent signals were observed with a confocal laser scanning microscope (Leica TCS SP8) 72 h after transformation.

### Co‐IP Assays

Rice protoplast expressing different constructs as indicated were extracted with Co‐IP buffer (50 mm, pH8.0 Tris‐HCl, 150 mm KCl, 1 mm EDTA, 0.5% TritonX‐100, 1 mm DTT, 1 mm PMSF, 1×Protease Inhibitor Cocktail (Roche)). After centrifugation at 12 000 rpm for 10 min, the supernatant was incubated with Flag beads (Sigma)/GFP beads (AlpalifeBio) for 4 h, and the beads were washed five times with wash buffer (50 mm, pH8.0 Tris‐HCl, 150 mm KCl, 1 mm EDTA, 0.5% TritonX‐100, 1 mm DTT, 1 mm PMSF, 1×Protease Inhibitor Cocktail (Roche)and 0.1% Nonidet P‐40). The proteins were eluted from the beads by boiling with 2×SDS sample buffer, and separated by SDS‐PAGE and immunoblotted with anti‐GFP (ABclonal, AE011, 1:5000 dilution) and anti‐Flag (ABclonal, AE005, 1:5000 dilution) antibodies.

### Physiological Measurements

The relative activity of PAL was measured according to the method described by He,^[^ ^22^ ^]^ with minor modification. ≈0.1 g 2‐week‐old seedlings were homogenized in 1 mL ice‐cold extraction (1 mm EDTA, 20 mm β‐mercaptoethanol, 1% PVP, and 0.1 M sodium borate buffer). The homogenate was centrifuged at 12 000 g for 20 min at 4 °C. The supernatant was used as the crude enzyme extract. 0.02 mL of supernatant (ice‐cold extraction as control (CK)) with 0.1 mL of 20 mm L‐phenylalanine and 0.28 mL of 0.1 mm sodium borate buffer (pH 8.8) was incubated for 30 min at 37 °C. The reaction was stopped by adding 0.02 mL 2 m HCl. The absorbance of the samples was recorded using a microplate reader (SpectraMax i3x, Molecular Devices) at a wavelength of 290 nm. The relative activity of PAL was calculated according to the formula (OD_290_(Samples)‐OD_290_(CK))/(OD_290_(WT)‐OD_290_(CK)). Lignin content and flavonoids content were measured using the kit G0708W and G0118W, respectively. The H_2_O_2_ and MDA contents were measured using kits G0168W48 and G0109W48, respectively, while the SOD and POD activities were measured using kits G0101W48 and G0107W48, respectively. All six kits were obtained from Grace Biotechnology.

### Semi‐In Vitro Ubiquitination Assays

Recombinant His‐PibH8CN was immobilized on Ni‐NTA beads (Thermo). Total proteins were extracted from leaves using ice‐cold extraction buffer (25 mm Tris‐HCl pH7.5, 10 mm NaCl, 10 mm MgCl_2_, 4 mm PMSF (freshly added), 5 mm DTT, 10 mm ATP, 100 µm MG132) followed by centrifugation at 15 000 × *g* for 20 min at 4 °C. Immobilized His‐PibH8CN complexes (50 µL beads volume) were incubated with clarified lysates from ZH11 (WT) or *FBK16*‐overexpressing (OE7) plants in reaction buffer (25 mm Tris‐HCl pH7.5, 150 mm NaCl, 0.5% (v/v) Triton X‐100, 1 mm PMSF, 2 mm DTT, 100 µm MG132) at 28 °C for 3 h rotation. Beads bound proteins were washed with increasing stringency buffer (25 mm Tris‐HCl pH 7.5, 300–600 mm NaCl, 0.5% (v/v) Triton X‐100, 1 mm PMSF, 2 mm DTT), and eluted by thermal denaturation in 1× Laemmli buffer (95 °C, 10 min) and resolved via 12% SDS‐PAGE. Ubiquitination and protein levels of PibH8CN were determined by Western blotting using a rabbit polyclonal anti‐ubiquitin antibody (1:1000; Cell Signaling Technology, #3936) and anti‐His antibody (ABclonal, AE003, 1:5000 dilution). Actin was used as a loading control (anti‐actin, 1:5000; Agrisera, #AS132640).

## Conflict of Interest

The authors declare no conflict of interest.

## Author Contributions

D.X., H.T., and Y.Y. contributed equally to this article. L.X. and F.D. designed the study. D.X., H.T., and Y.Y. contributed equally to data collection and drafted the manuscript. Y.Y. and Y. Y. constructed the NILs. D.X., H.T., Y.Y., H.W., W.L., Y.W., Q.L., Y.C., H.H., and D.L. collected data and performed analysis. H.H. and M.Y. provide technical support. X.L., H.X., F.D., and L.X. revised the manuscript. All authors read and approved the contents of this paper.

## Supporting information



Supporting Information

Supplemental Table 1–4

## Data Availability

The data that support the findings of this study are available from the corresponding author upon reasonable request.
