# Supplementary material for: A Blast‐Resistant NLR Gene Confers Drought Resistance by Competitively Interacting with an E3 Ligase to Protect Phenylalanine Ammonia‐Lyase in Rice
Source: Adv Sci (Weinh). 2025 Jul 21;12(39):e02662. doi: 10.1002/advs.202502662 (PMC12533315; doi:10.1002/advs.202502662)
Supplement: Supplementary file 1 — Supporting Information [file ADVS-12-e02662-s002.docx]

**Supplemental Figures**


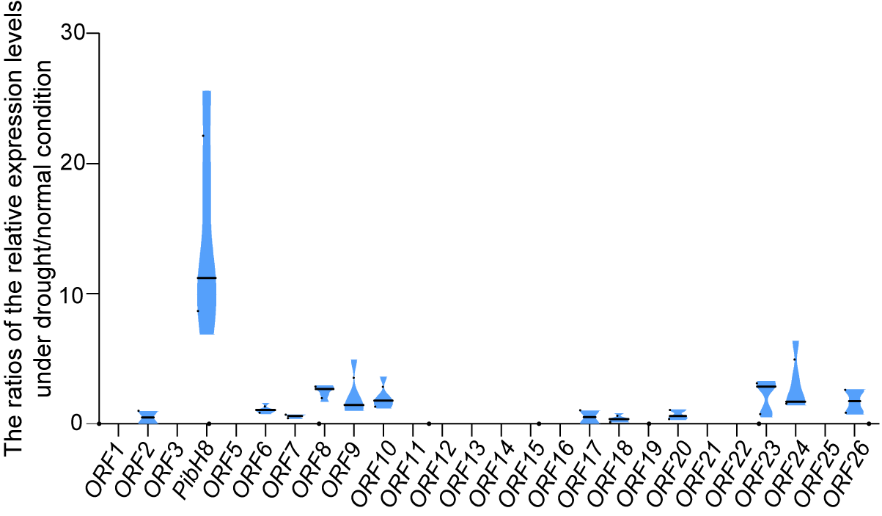


Figure S1. The ratios of the relative expression levels of 26 genes under drought condition to those under normal condition.


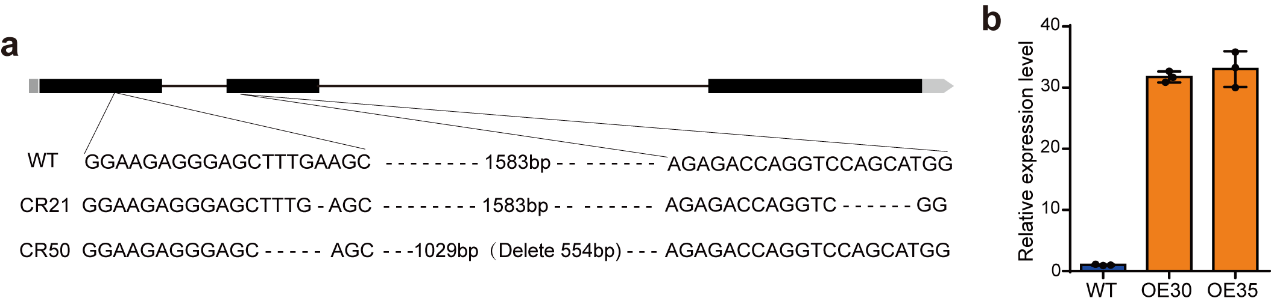


Figure S2. Identification of *PibH8*-positive transgenic lines. a) Gene structure and mutation sites in *PibH8*. b) Expression levels of *PibH8* in *PibH8-*overexpression lines at the seedling stage.


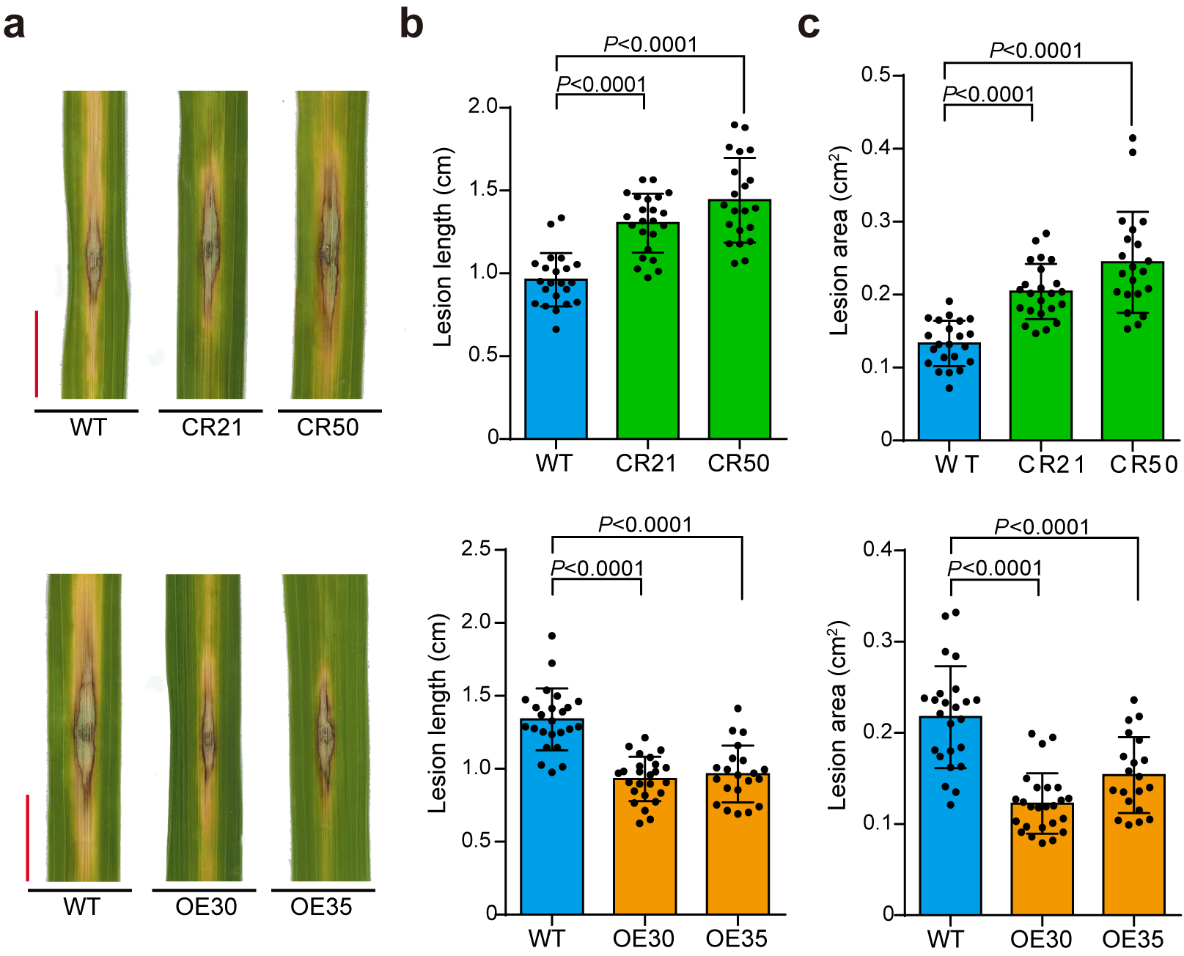


Figure S3. The blast disease resistance performance of *PibH8* mutant lines and overexpressing lines. a) Images of *PibH8* mutant lines and overexpressing lines compared with WT after inoculation with blast fungus (*Magnaporthe oryzae*, *TLP37*). Scale bars, 1 cm. b) The lesion length of *PibH8* mutant lines and overexpressing lines compared with WT after inoculation with blast fungus. Data represent means ± SD (*n* ＞ 15). Significance was determined by Student’s *t*-test. c) The lesion area of *PibH8* mutant lines and overexpressing lines compared with WT after inoculation with blast fungus. Data represent means ± SD (*n* ＞ 15). Significance was determined by Student’s *t*-test.


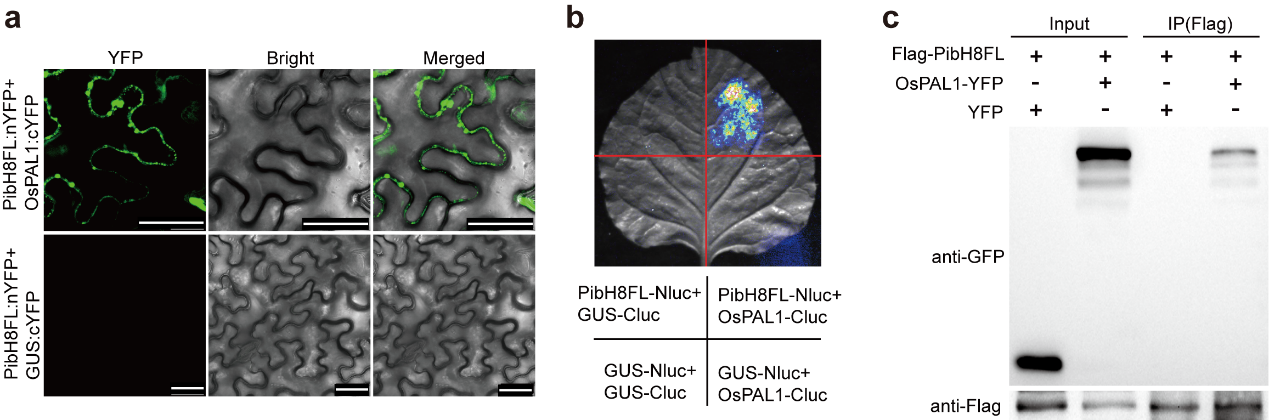


Figure S4. Identification of the interaction between OsPAL1 and PibH8FL. a) BiFC assay shows the interaction of PibH8FL and OsPAL1. GUS:cYFP served as a negative control. Scale bars, 50 µm. b) SFLC assay shows the interaction of PibH8FL and OsPAL1. An unrelated protein GUS served as a negative control. c) Interaction confirmation of PibH8FL and OsPAL1 by Co-IP in rice protoplasts. Crude protein extracts from stably expressed OsPAL1-YFP and Flag-PibH8FL or YFP and Flag-PibH8FL were used as input. Input proteins were immunoprecipitated with Flag-beads. The input and co-immunoprecipitated proteins were detected with anti-GFP and anti-Flag antibodies as indicated.


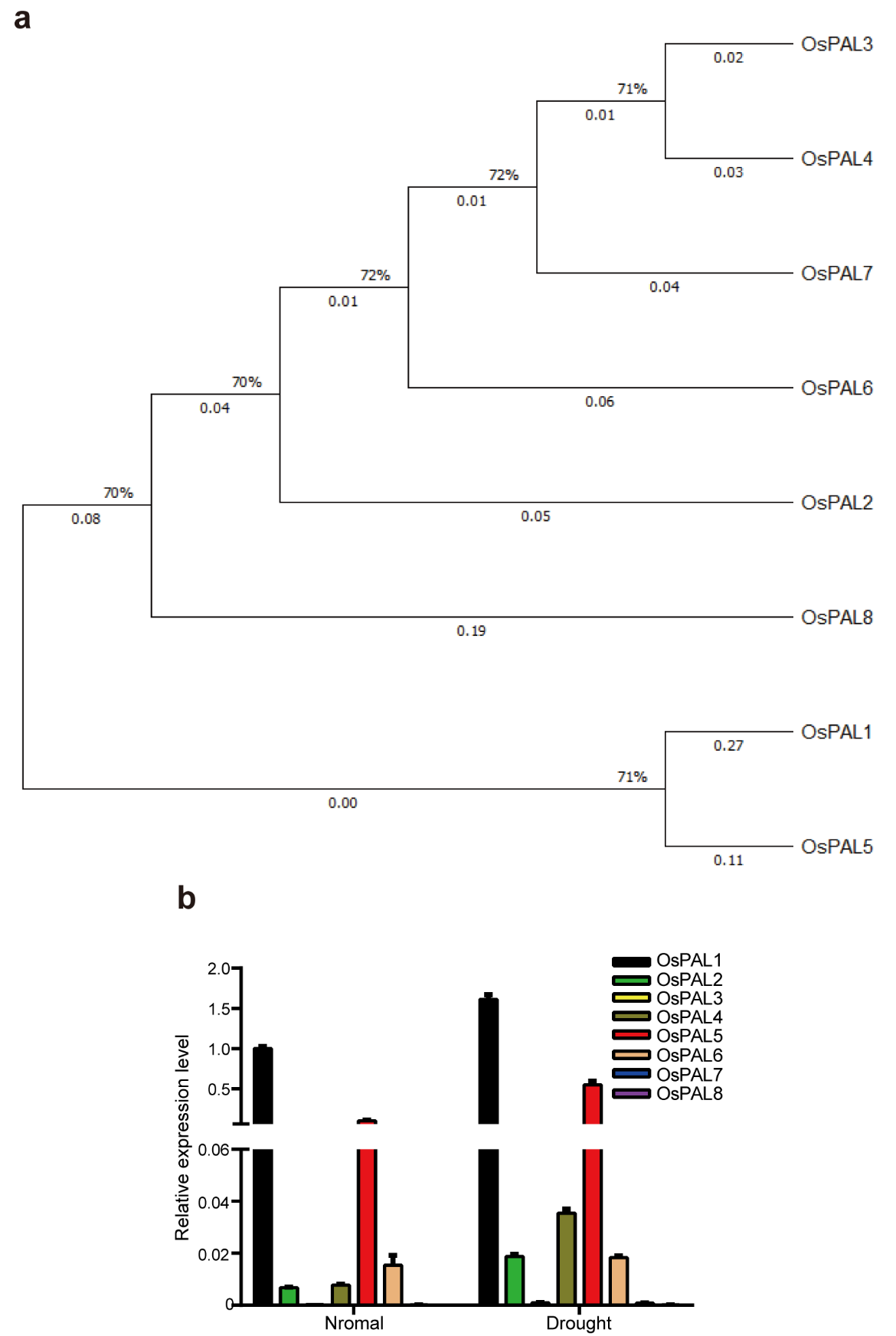


Figure S5. Phylogenetic analyses of the PAL family in rice and drought-responsiveness analyses of the PAL family and *PibH8*. a) The phylogenetic tree of PAL family genes in rice. b) The expression levels of PAL family genes in rice seedlings under normal and drought-stressed conditions. Data represent means ± SD (*n* = 3).


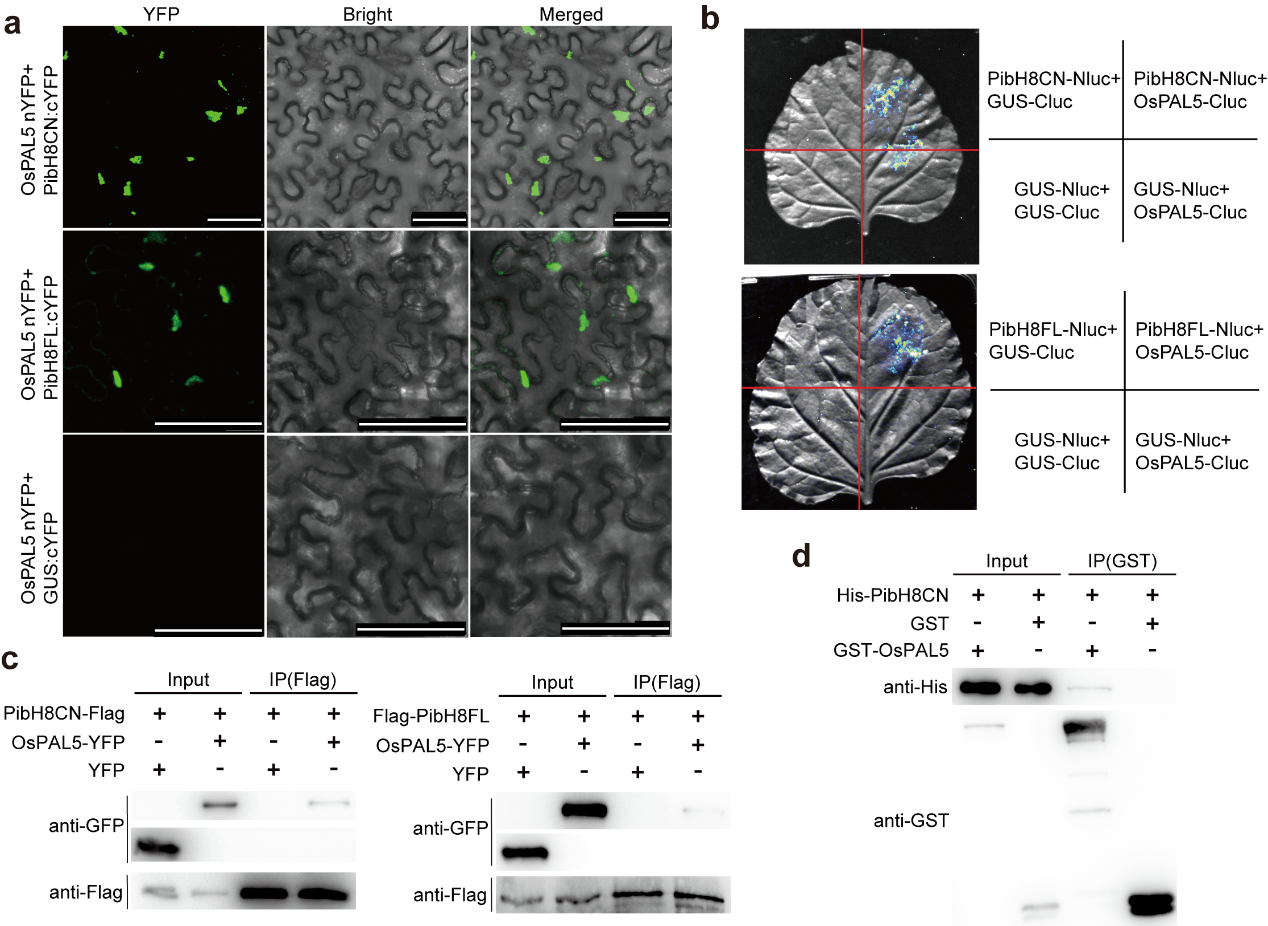


Figure S6. The interaction analysis of PibH8CN/FL with OsPAL5. a) Confirmation of the interaction of PibH8CN/FL with OsPAL5 by BiFC assays in tobacco. Scale bars, 50 µm. GUS:cYFP served as a negative control. b) Confirmation of the interaction of PibH8CN/FL with OsPAL5 by SFLC assays in tobacco. c) Confirmation of the interaction of PibH8CN/FL with OsPAL5 by Co-IP assays in in rice protoplasts. OsPAL5-YFP and Flag-PibH8CN/FL or YFP and Flag-PibH8CN/FL were used as input. Input proteins were immunoprecipitated with Flag-beads. The input and co-immunoprecipitated proteins were detected with anti-GFP and anti-Flag antibodies as indicated. d) Pull‐down assays showed the physical interaction of PibH8CN and OsPAL5. His-PibH8CN, GST-OsPAL5, and GST were expressed in *E*. *coli* strain BL21 (DE3). GST-OsPAL5 or GST was incubated with His-PibH8CN and IP with glutathione agarose. Input and pull-downed proteins were detected with anti-GST or anti-His antibodies as indicated.


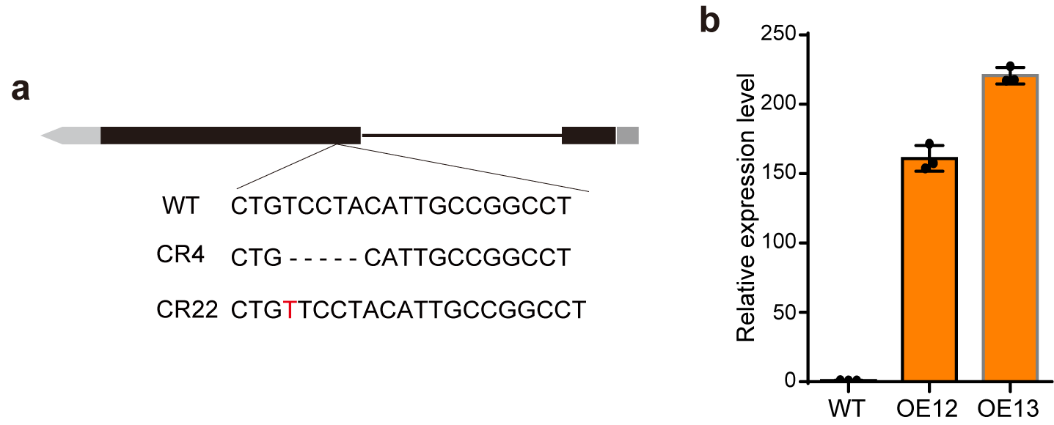


Figure S7. Identification of *OsPAL1* transgenic lines. a) Gene structure and mutation sites in *OsPAL1*. b) Expression levels of *OsPAL1* in *OsPAL1-*overexpression lines at seedling stage. Data represent means ± SD (*n* = 3).


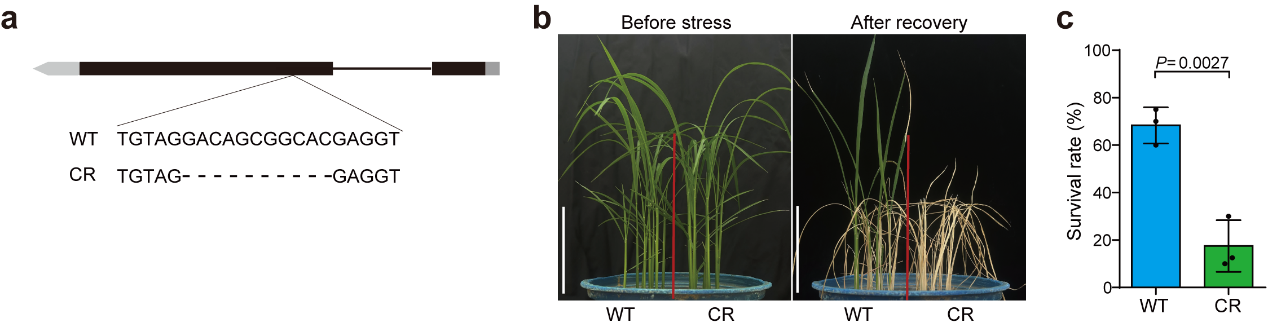


Figure S8. Identification of the genotype and drought resistance of *OsPAL5* CRISPR mutant. a) Gene structure and mutation site in *OsPAL5*. b) Plant performance of *OsPAL5* mutant (CR) and WT before and after recovery from drought stress at the seedling stage. Scale bars, 10 cm. c) Survival rates of *OsPAL5* mutant (CR) and WT after drought stress treatment. Data represent means ± SD (*n* = 3). Significance was determined by Student’s *t*-test.


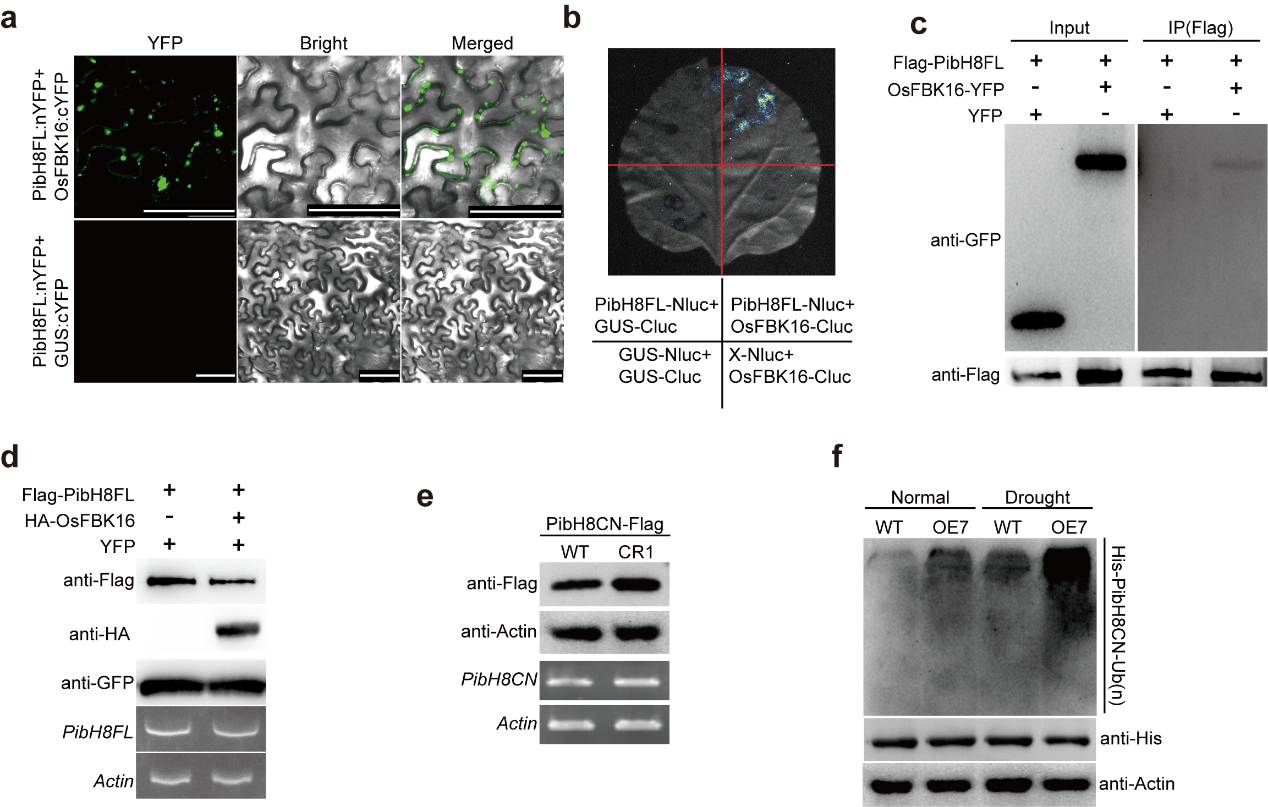


Figure S9. OsFBK16 interacts with and degrades PibH8FL. a) BiFC assay shows the interaction of PibH8FL and OsFBK16. GUS:cYFP served as a negative control. Scale bars, 100 µm. b) SFLC assay shows the interaction of PibH8FL and OsFBK16. An unrelated protein GUS served as a negative control. c) Interaction confirmation of PibH8FL and OsFBK16 by Co-IP in rice protoplasts. d) Co-transformation assay in rice protoplasts shows that HA-OsFBK16 can degrade Flag-PibH8FL. Proteins were detected with Flag, HA or GFP antibody by Western blot, and the transcription levels of *PibH8FL* and *Actin* as internal control was detected by RT-PCR. e) Transformation assay in rice protoplasts shows that the protein accumulation level of PibH8CN-Flag was significantly higher in *OsFBK16*-CR1 than in the wild-type control. Proteins were detected with Flag or Actin antibody by Western blot, and the transcription levels of *PibH8CN* and *Actin* as internal control was detected by RT-PCR. f) Semi-*in vitro* ubiquitination assays. The His-PibH8CN protein was incubated with glutathione beads at 4°C for 2 h, then was incubated with equal amounts of protein extracts from 4-week-old WT and *OsFBK16*-OE7 plants under normal and drought conditions at 28°C for 3 h. Samples were subjected to immunoblot analysis with anti-Ub (top panels), anti-His (middle panels) and anti-Actin (bottom panels) antibody.


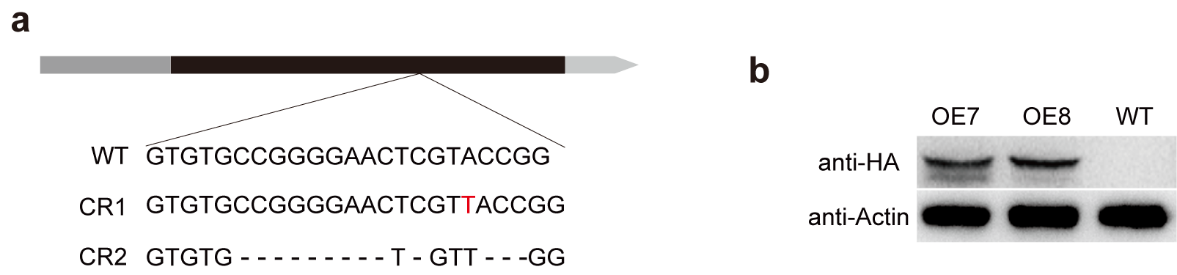


Figure S10. Identification of *OsFBK16* transgenic lines. a) Gene structure and mutation sites (by CRISPR-Cas9) in *OsFBK16*. b) Western blot of HA-OsFBK16 protein levels in the HA-*OsFBK16*-overexpression lines and WT using HA antibody at the seedling stage.


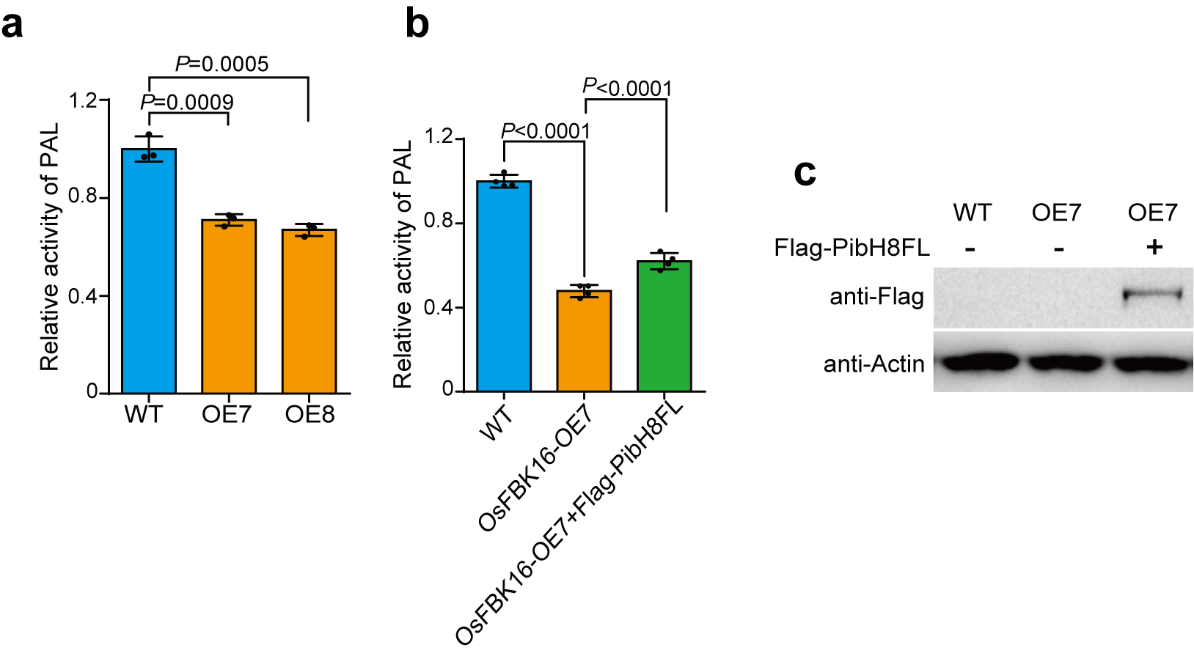


Figure S11. PibH8 can partially rescue the reduced relative activity of PAL caused by OsFBK16. a) Relative activity of PAL in *OsFBK16* overexpression lines compared to WT. Data represent means ± SD (*n* = 3). Significance was determined by Student’s *t*-test. b) Relative activity of PAL of WT, *OsFBK16*-OE7, and *OsFBK16*-OE7 + Flag-PibH8FL in rice protoplasts. Data represent means ± SD (*n* = 4). Significance was determined by Student’s *t*-test. c) Detection of PibH8FL and Actin proteins of the samples in (b).


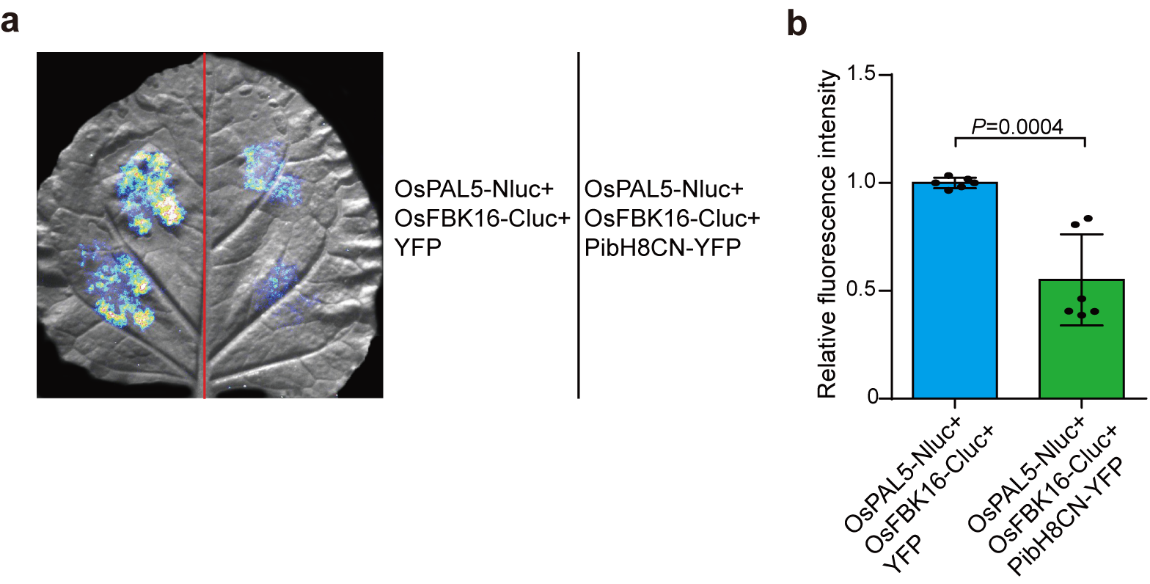


Figure S12. PibH8 enhances the protein stability of OsPAL5 by competitively binding to OsFBK16. a) Competition SFLC assay in tobacco showed that PibH8CN could inhibit the interaction between OsFBK16 and OsPAL5, and YFP was used as the negative control. b) Statistical analysis of relative fluorescence intensity in the competition SFLC assay. PibH8CN-YFP significantly reduced the fluorescence intensity of OsPAL5-OsFBK16 interaction compared to YFP. Data represent means ± SD (*n* = 6). Significance was determined by Student’s *t*-test.


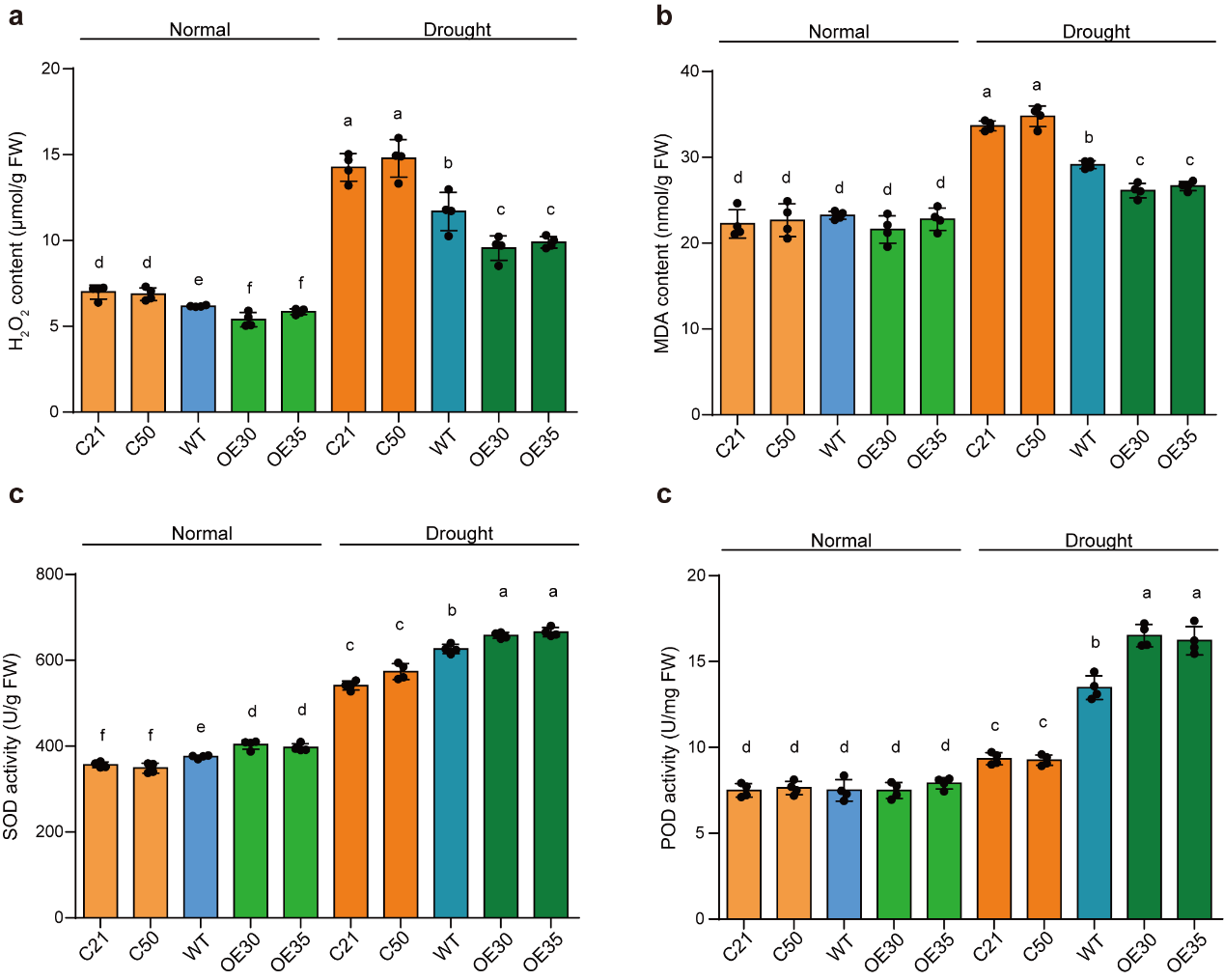


Figure S13. Examination of the H_2_O_2_ (a) and MDA (b) content, as well as the SOD (c) and POD (d) activity in the *PibH8* transgenic lines. Data represent means ± SD (*n* = 4). Significance was determined by Student’s *t*-test. The letter a, b, c, d, e or f above the bar indicates a significant difference at *P* < 0.05.


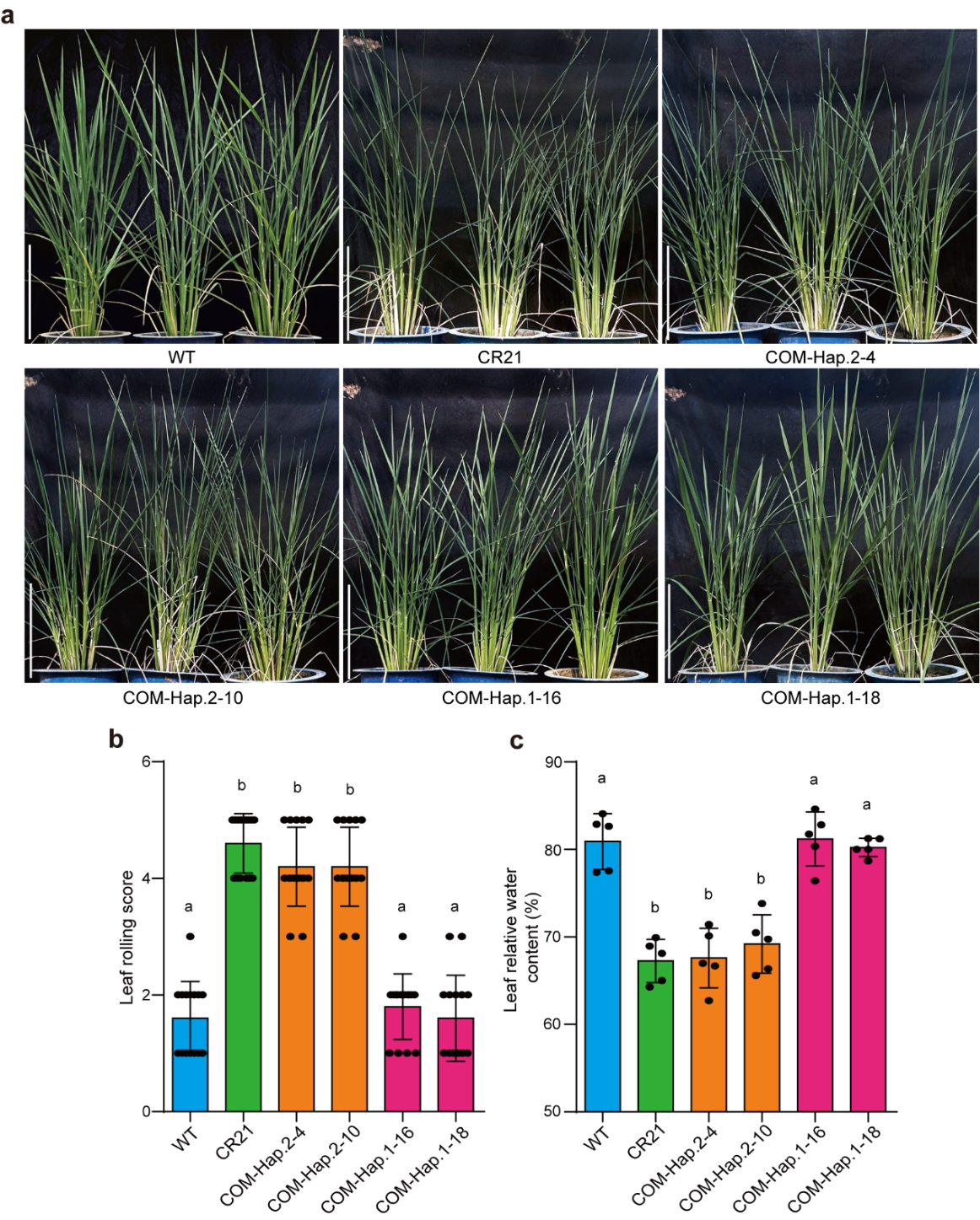


Figure S14. Drought resistance performance of two haplotype complementary lines of *PibH8* at panicle development stage. a) Images of two haplotypes complementary materials of *PibH8* under drought stress at panicle development stage. Scale bars, 20 cm. b) Comparative analysis of leaf rolling score of two complementation lines (*PibH8*-Hap.1 and *PibH8*-Hap.2), *PibH8*-mutant (CR21), and WT under drought stress. Data represent means ± SD (*n* = 15). Significance was determined by one-way ANOVA with Tukey’s test. c) Comparison analysis of leaf relative water content of two complementation lines, *PibH8*-CR21, and WT under drought stress. Data represent means ± SD (*n* = 5). Significance was determined by one-way ANOVA with Tukey’s test.


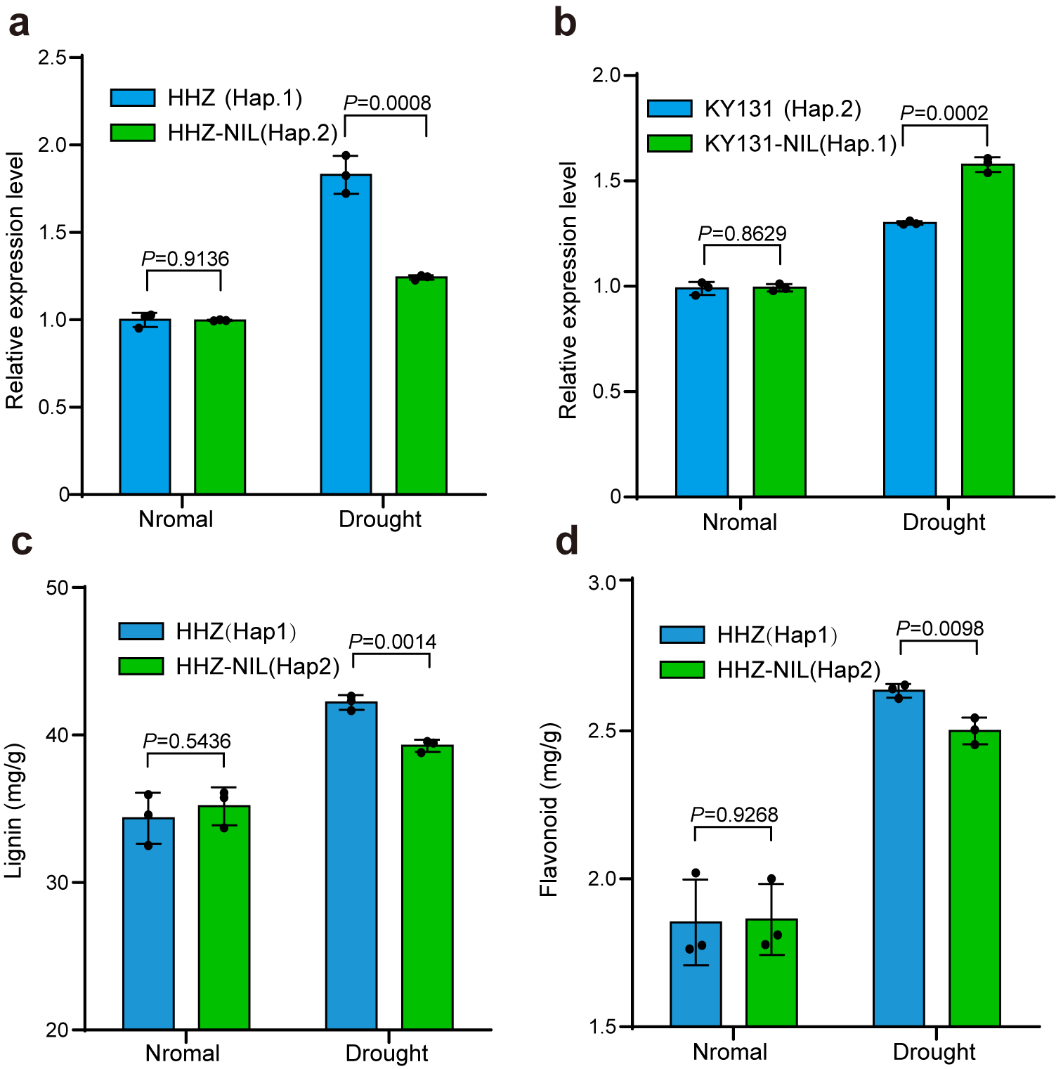


Figure S15. Determination of the relative expression level and the content of lignin and flavonoids in *PibH8* NIL. a) The relative expression of *PibH8* in HHZ (Hap.1) and HHZ-NIL (Hap.2) under normal and drought conditions. Data represent means ± SD (*n* = 3). b) The relative expression of *PibH8* in KY131 (Hap.2) and KY131-NIL (Hap.1) under normal and drought conditions. Data represent means ± SD (*n* = 3). c) Lignin content of HHZ (Hap.1) and HHZ-NIL (Hap.2) under normal drought conditions. Data represent means ± SD (*n* = 3). d) Flavonoids content of HHZ (Hap.1) and HHZ-NIL (Hap.2) under normal drought conditions. Data represent means ± SD (*n* = 3). The significance of all the above data was determined by Student’s *t*-test.


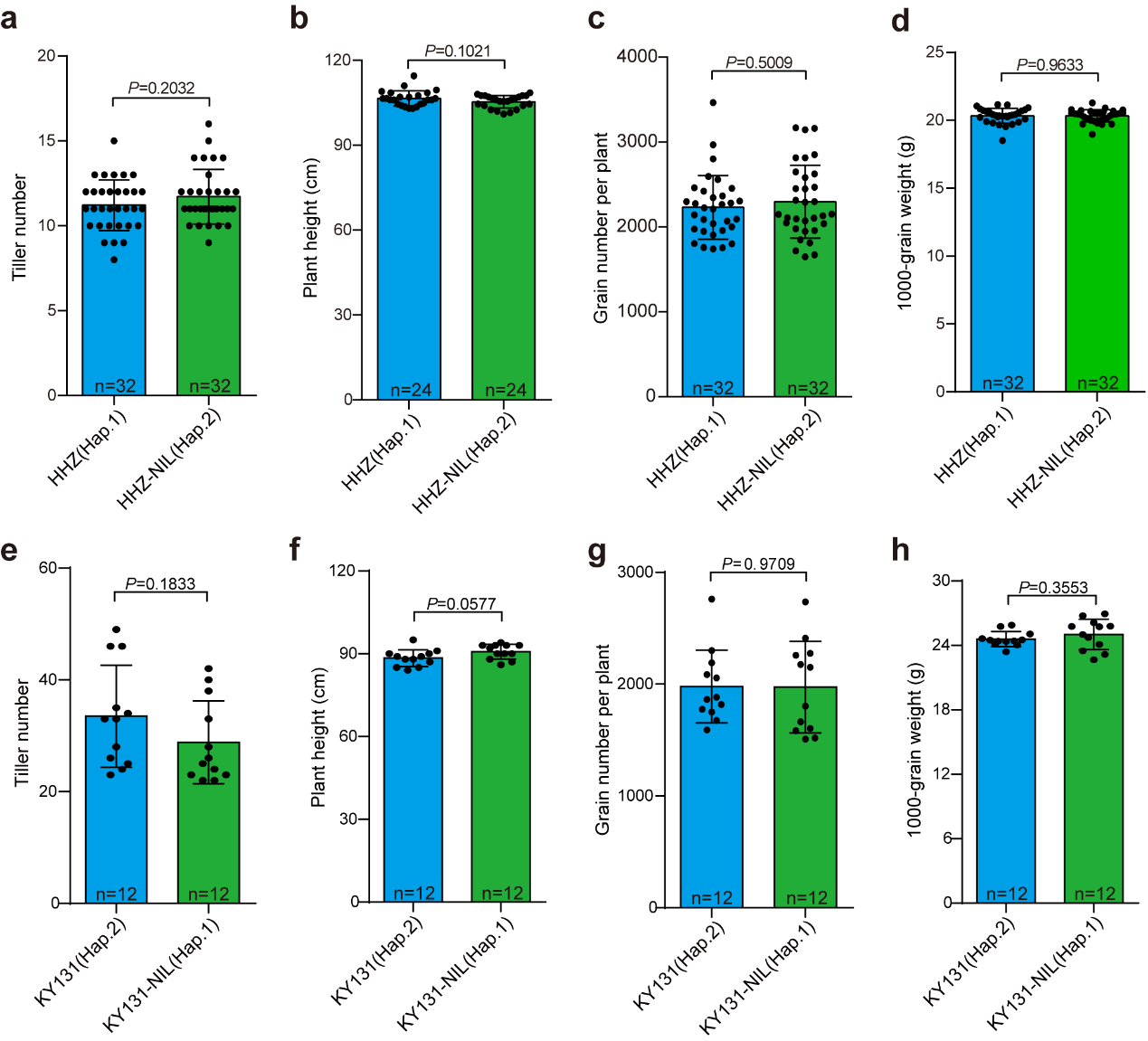


Figure S16. Examination of agronomic traits of *PibH8* NILs under normal conditions. a-d) Examination of tiller number, plant height, grain number of per plant and 1000-grain weight of *PibH8* NIL in HHZ background under normal condition. Data represent means ± SD (*n* ≥ 20). e-h) Examination of tiller number, plant height, grain number per plant and 1000-grain weight of *PibH8* NIL in the KY131 background under normal condition. Data represent means ± SD (*n* = 12). The significance of all the above data was determined by Student’s *t*-test.


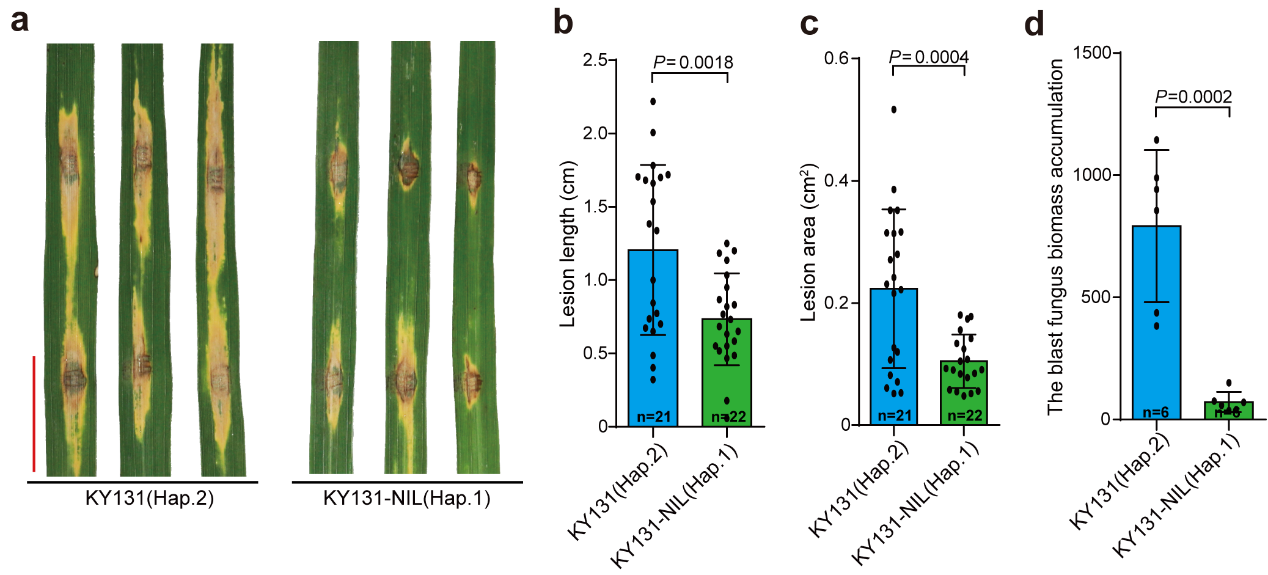


Figure S17. The rice blast disease resistance performance of KY131 (Hap.2) and KY131-NIL (Hap.1). a) Images of KY131 (Hap.2) and KY131-NIL (Hap.1) after inoculation with blast fungus (*Magnaporthe oryzae*, *YC6*). Scale bars, 1 cm. b) The lesion length of KY131 (Hap.2) and KY131-NIL (Hap.1) after inoculation with blast fungus. Data represent means ± SD (*n*＞20). c) The lesion area of KY131 (Hap.2) and KY131-NIL (Hap.1) compared with WT after inoculation with blast fungus. Data represent means ± SD (*n*＞20). d) The blast fungus biomass accumulation on KY131 (Hap.2) and KY131-NIL (Hap.1) leaves after inoculation. Data represent means ± SD (*n*=6). The significance of all the above data was determined by Student’s *t*-test.
